# Integrative Multi-Omics Analysis Provides Insights into Floral Organ Development and Flower Color Formation in *Renanthera coccinea*, a Rare Tropical Orchid

**DOI:** 10.3390/biology15171497

**Published:** 2026-09-02

**Authors:** Xin Yang, Xia Lin, Xiaoyang Wang, Mengpei Shi, Lina Sang, Lang Zhang, Sai Huang, Jingfei Wang, Xuan Chen, Jun Yang, Chunyu Weng, Dingmin Liang, Huilin Zhao, Junfang Ren, Huiqi Zhao

**Affiliations:** 1 Sanya Institute, Hainan Academy of Agricultural Sciences, Sanya 572025, China; xiny55690@gmail.com (X.Y.); linxia23400@163.com (X.L.); 13075358645@163.com (X.W.); simengpei@outlook.com (M.S.); sanglina@foxmail.com (L.S.); juny83@126.com (J.Y.); 2 The Key Laboratory of Tropic Special Economic Plant Innovation and Utilization, National Germplasm Resource Chengmai Observation and Experiment Station, Institute of Tropical Horticulture Research, Hainan Academy of Agricultural Sciences, Haikou 571100, China; zlang123@126.com (L.Z.); huangsai512@163.com (S.H.); wangjf5821@163.com (J.W.); chenxuan06@sina.com (X.C.); chunyuminmin@163.com (C.W.); m13307588828@163.com (D.L.); 3 School of Tropical Agriculture and Forestry, Hainan University, Haikou 571100, China; 4 Plant Protection and Quarantine Center of Shanxi Province, Taiyuan 030000, China; zhaohuilin666@163.com

**Keywords:** *Renanthera*, floral organ identity, lip specialization, flower color patterning

## Abstract

The genus *Renanthera* is valued for its fiery-red floral coloration and distinctive spider-like floral architecture; however, the molecular mechanisms underlying floral organ specialization and perianth color differentiation remain poorly understood. In this study, using integrated morphological, transcriptomic, and metabolomic analyses of *R. coccinea*, we found that the cell types of the dorsal and lateral perianth organs were broadly similar but clearly distinct from those of the lip and the column. Developmentally, the organ-specific expression patterns of *AGAMOUS-LIKE6-1/2* (*AGL6-1/2*), *APETALA3-1/2* (*AP3-1/2*), and *AGAMOUS* (*AG*) were consistent with the conserved orchid floral organ identity model. Furthermore, lip-enriched expression of brassinosteroid-responsive RING H2 (*BRH1*) and flavin-containing monooxygenase *YUCCA10* (*YUC10*) suggests that brassinosteroid- and auxin-associated pathways may contribute to lip specialization, especially spur development. Regarding pigmentation, high expression of flavonoid 3′-hydroxylase (*F3′H*) was associated with a cyanidin-dominated anthocyanin profile. The color divergence among the dorsal sepal, petals, and lateral sepals was associated with differential expression of UDP-glucose: flavonoid 3-O-glucosyltransferase (*UFGT*), which was co-expressed with plant U-box protein 9 (*PUB9*) and carbon catabolite repressor 4 (*CCR4*), associated with distinct patterns of pigment modifications. These findings provide a molecular framework for understanding floral organ development and color patterning in *R. coccinea* and candidate genes for future validation and molecular breeding in *Renanthera*.

## Simple Summary

The Flame Orchid (*Renanthera coccinea*) is prized for its striking red color and unique spider-like flowers, yet how its distinct shapes and color patterns form remains mysterious. By analyzing the phenotypic traits, genes, and chemical compounds of its flowers, we uncovered key regulators potentially controlling flower shape and specialized structures like the spur. We also identified specific enzymes that modify red pigments, creating color differences among sepals. These findings reveal how complex orchid flowers develop and provide valuable genetic targets to help breeders create unique ornamental orchid varieties.

## 1. Introduction

*Renanthera* Lour. (Flame Orchid), a genus within the subtribe Aeridinae (Vandeae, Orchidaceae), comprises approximately 20 species of perennial epiphytic or lithophytic herbs distributed mainly from the eastern Himalayas to Southeast Asia [1]. In China, three species have been recorded: *R. citrina*, *R. coccinea*, and *R. imschootiana*, the latter two of which are listed as Endangered (EN) and Critically Endangered (CR), respectively, in the Red List of China’s Higher Plants [2,3,4]. The zygomorphic flowers of *Renanthera* consist of one dorsal sepal, two lateral sepals, two petals, a spurred lip (labellum), and a column (gynostemium) [1,5]. Known for their spectacular branched inflorescences and long-lasting blooms, these orchids are characterized by a signature vermilion red color, although genetic studies suggest that yellow is the genuine base hue [1]. As a core genetic source for red hybrids in the Southeast Asian cut-flower industry, *Renanthera* holds an irreplaceable position in landscape horticulture and molecular breeding [1,6]. Therefore, deciphering the molecular mechanisms underlying floral development and pigment biosynthesis will deepen our understanding of floral organ identity and morphological diversification from an Evolutionary Developmental Biology (Evo-Devo) perspective and provide a theoretical basis for the precision breeding of floral shape and color in *Renanthera*.

The ABCE and floral symmetry models, derived largely from mutant studies in model species such as *Arabidopsis thaliana* and snapdragon (*Antirrhinum majus*), provide a fundamental framework for understanding the developmental basis of floral diversity [7,8,9]. According to these models, sepal identity is specified by the combination of A- and E-function genes, while petals, stamens, and carpels are determined by A + B + E, B + C + E, and C + E gene complexes, respectively [10]. Notably, except for *AP2*, an A-function gene belonging to the *AP2/ERF* family [11], nearly all well-characterized floral organ identity genes encode MADS-box transcription factors [12,13]. The determination of bilateral symmetry (zygomorphy) is primarily orchestrated by the *CYCLOIDEA* (*CYC*) and *DICHOTOMA* (*DICH*) genes within the TCP family, alongside the MYB family genes *RADIALIS* (*RAD*) and *DIVARICATA* (*DIV*). Specifically, *CYC/DICH* and *RAD* direct dorsal identity, whereas *DIV* governs lateral differentiation [14,15,16,17]. Building on these models, comparative studies across angiosperm lineages have shown that changes in the spatiotemporal expression of floral identity and symmetry genes contribute substantially to the diversification of floral architecture [18,19,20,21,22]. In Orchidaceae, the diversification of floral structures is specifically interpreted through the “Orchid Code”, “Homeotic Transformation (HOT)”, and “Perianth code (P-code)” models, which are rooted in the duplication and functional divergence of the *AP3* and *AGL6* lineages [23,24,25]. Recent advances have further identified specific regulators of lip specialization: *Phalaenopsis aphrodite WUSCHEL-related homeobox 3 (PaWOX3)* and *AGL6-2* function redundantly in orchestrating specialized lip structures [26], whereas the *P. equestris* Auxin-Associated F-box protein (*PeAAF*) gene facilitates labellum expansion by amplifying auxin responsiveness [27]. Additionally, *P. equestris* NAC domain-containing protein 67 (*PeNAC67*) and *P. equestris* KANADI 2 (*PeKAN2*) have been implicated in promoting labellum traits [28], further highlighting the complex and potentially lineage-specific mechanisms governing lip differentiation. Moreover, the regulatory programs dictating bilateral symmetry appear to be evolutionarily divergent among orchid genera. While the dorsal expression of three *CYC* genes mediates zygomorphy in *Phalaenopsis* [29], symmetry in *Cymbidium sinense* is predominantly governed by Class B and Class G genes [30]. Such discrepancies suggest that the developmental pathways for bilateral symmetry have undergone lineage-specific evolution within the Orchidaceae family.

The mechanisms of floral pigmentation are primarily centered on four major classes of pigments: chlorophylls, carotenoids, anthocyanins, and betalains [31,32]. Chlorophylls confer green color to cells; carotenoids produce yellow, orange, or red hues; while anthocyanins and betalains provide a broad spectrum ranging from purple to blue and orange to red, respectively [32,33,34]. Except for betalains, which are largely restricted to Caryophyllales and are mutually exclusive with anthocyanins, these pigment classes are widely distributed across higher plants. The key structural genes regulating the biosynthesis of these pigments are highly conserved throughout evolution [31,32,35]. Despite this genetic conservation, the mechanisms responsible for diverse color patterns are complex and varied. For instance, in the blue/purple *Ipomoea purpurea*, pink and red flowers have emerged; the former results from a mutation in the coding region of the structural gene flavonoid 3′-hydroxylase (*F3′H*) [36,37], while the latter is caused by the downregulation of *F3′H* expression [36,38,39]. Notably, to date, the formation of irregular patches on floral organs has been attributed to the insertion of transposons into structural genes. For example, variegated phenotypes in *I. purpurea* and *Torenia fournieri* are driven by transposon insertions within structural genes and subsequent partial reversion mutations [40,41]. Recent multi-omics studies in orchids, such as the GA–ABA regulatory hub identified in *Cymbidium sinense* [42] and the MYB-mediated lip coloration in *Cymbidium floribundum* [43] have significantly advanced our understanding of orchid development. Despite these advancements, the regulatory mechanisms governing complex intra-floral color divergence remain largely unknown, especially regarding the formation of specific irregular patterns versus monochromaticity among different floral organs.

Unlike previous studies focusing on broad developmental stages or uniform coloration, in the present study, we used *R. coccinea* as a representative species and integrated morphological, anatomical, transcriptomic, and metabolomic approaches to investigate floral organ development and pigmentation. Our objectives were to: (1) characterize the micromorphological variations in epidermal cells among different floral organs; (2) elucidate the developmental programs of the flower, specifically the mechanisms governing lip specialization; (3) identify the key pigment constituents responsible for the irregular spotting on dorsal sepal and petals versus the monochromatic coloration of lateral sepals; and (4) explore the regulatory networks underlying the divergent pigmentation patterns observed between dorsal and lateral floral organs.

## 2. Materials and Methods

### 2.1. Plant Materials and Growth Conditions

Living wild-sourced specimens of *R. Coccinea* were maintained at the Orchid Germplasm Resource Nursery of the Hainan Academy of Agricultural Sciences (Haikou, China). The plants were cultivated in an open-sided greenhouse under natural sunlight and a typical tropical monsoon climate, with ambient temperatures ranging from 25–32 °C and a relative humidity of ~80%. The potting substrate consisted of a mixture of limestone gravel, pine bark, and expanded clay pellets. Under these conditions, *R. coccinea* typically flowers from March to May, with the blooming duration lasting approximately three months.

### 2.2. Morphological and Micromorphological Observations

For stereomicroscopic photography, various floral organs of *R. coccinea* were dissected and photographed using a Nikon Model CDSS230 stereomicroscope equipped with a Nikon DXM1200F digital camera (Nikon Instech Co., Ltd., Kawasaki, Japan). For scanning electron microscopy (SEM), floral organs from mature flowers were fixed in fresh FAA (3.7% formaldehyde, 5% acetic acid, and 50% ethanol), followed by dehydration in a graded ethanol series and drying with a CO_2_ critical-point dryer. After being sputter-coated with gold, the samples were examined using a Hitachi S-4800 scanning electron microscope (Hitachi High-Technologies Corp., Tokyo, Japan).

### 2.3. Reference Transcriptome Assembly and DGE Profiling

To generate a comprehensive reference transcriptome for *R. coccinea*, total RNA was extracted from various tissues (buds, leaves, stems, and roots) across different developmental stages, pooled in equal amounts, and subjected to hybrid sequencing using both PacBio Sequel II and Illumina HiSeq 2000 platforms. PacBio long-read sequencing yielded 90,145 full-length non-chimeric (FLNC) reads totaling 306,141,234 bp (N50: 3536 bp; N90: 2372 bp; GC content: 41.37%). Illumina short reads were de novo assembled using Trinity [44], generating 440,693 transcripts with a total length of 597,692,166 bp (N50: 1748 bp; N90: 636 bp; GC content: 38.29%). The Illumina-derived transcripts were integrated with PacBio FLNC reads, clustered to represent different isoforms via all-by-all BLASTN (https://ftp.ncbi.nlm.nih.gov/blast/executables/blast+/LATEST/; accessed on 10 October 2025) searches [45], and deduplicated using CD-HIT with a sequence identity threshold of 0.95 to yield 122,032 non-redundant unigenes (total length: 135,625,900 bp; N50: 2086 bp; N90: 690 bp; GC content: 42.53%). Protein-coding sequences (CDS) were predicted using ANGEL, filtering for sequences longer than 100 amino acids. Functional annotation of unigenes was performed using BLASTX (E-value < 1 × 10^−5^) against the *Arabidopsis thaliana* CDS database in TAIR. Transcriptome completeness evaluated by BUSCO (Benchmarking Universal Single-Copy Orthologs) revealed that among 1614 conserved orthologs, complete genes accounted for 89.5% (1412 single-copy and 33 duplicated genes), while fragmented and missing genes accounted for 6.4% (104) and 4.0% (65), respectively. These metrics demonstrated high assembly completeness, confirming the suitability of the reference transcriptome for downstream expression profiling.

To profile gene expression dynamics across floral organs and developmental stages, floral tissues were harvested at two key stages: the small stage (S; marking the completion of organ differentiation and the onset of coloration) and the middle stage (M; representing the full maturation of floral pigmentation). Five distinct floral organs were sampled per stage: the petal (pe), dorsal sepal (ds), lateral sepal (ls), lip (lp), and column (co). At each of the two developmental stages, five distinct floral organs were sampled, with three independent biological replicates collected for each organ, resulting in a total of 30 RNA samples. Total RNA was extracted using the SV Total RNA Isolation System (Madison, WI, USA) according to the manufacturer’s instructions. Following quality control, 30 independent transcriptome libraries were constructed and subjected to 150-bp paired-end sequencing on the Illumina HiSeq platform (Novogene, Beijing, China).

Raw reads were quality-filtered to obtain clean reads, which were subsequently aligned to the *R. coccinea* reference transcriptome using Bowtie2 [46]. The total amount of clean data was approximately 195.56 Gb. Gene expression levels were quantified as raw counts and Fragments Per Kilobase of transcript per Million mapped reads (FPKM) using RSEM [46]. Principal Component Analysis (PCA) was conducted on the FPKM matrix using the prcomp function in R (v4.3.1) to evaluate overall sample relationships and biological replicate consistency. Differentially expressed genes (DEGs) between pairwise conditions were identified from the raw count matrix using DESeq2. Raw counts were normalized using the median-of-ratios method. DESeq2 estimates expression dispersion via a negative binomial distribution model and evaluates expression differences using Wald tests, with PP-values adjusted for multiple testing via the Benjamini–Hochberg method. The criteria for DEGs were set as ∣log_2_ FC∣ ≥ 1 and *P*_adj_ < 0.05 [46]. Volcano plots were generated to visualize DEG distributions, where red, blue, and gray points represent significantly up-regulated, down-regulated, and non-significantly expressed genes, respectively. For narrative simplicity and consistency with standard transcriptomic literature, these non-redundant unigenes generated from the de novo assembly are referred to as ‘genes’ (and ‘DEGs’ for differentially expressed unigenes) throughout the manuscript.

### 2.4. Generation of the Metabolomic Datasets and DAM Profiles

Metabolomic profiling was performed on dorsal sepals (ds) and lateral sepals (ls) collected at the same two developmental stages (S and M) as the transcriptomic samples, using three independent biological replicates per sample group. Sample extraction was carried out following the method described by Zhang et al. [47]. with minor modifications. Briefly, freeze-dried tissue samples were ground into a fine powder, and metabolites were extracted using pre-chilled LC-MS-grade methanol (Thermo Fisher Scientific, Waltham, MA, USA). Following low-temperature centrifugation, the resulting supernatant was collected for LC-MS/MS analysis. All reagents, including water, formic acid, and acetonitrile, were of LC-MS grade (Thermo Fisher Scientific). Metabolomic profiling services were conducted by Novogene Co., Ltd. (Beijing, China).

Chromatographic separation was achieved using an ExionLC ultra-performance liquid chromatography (UPLC) system coupled with a QTRAP^®^ 6500+ mass spectrometer (SCIEX, Framingham, MA, USA). Separation was performed on an XSelect HSS T3 column (Waters, Milford, MA, USA; 2.1 × 150 mm, 2.5 μm) at a flow rate of 0.4 mL/min. Mobile phase A consisted of 0.1% formic acid in water (*v*/*v*), and mobile phase B comprised 0.1% formic acid in acetonitrile (*v*/*v*). The gradient elution program was configured as follows: 0–2 min, 2% B; 2–15 min, linear gradient from 2% to 100% B; 15–17 min, 100% B; 17–17.1 min, linear gradient from 100% to 2% B; and 17.1–20 min, 2% B. The QTRAP^®^ 6500+ mass spectrometer was operated in both positive and negative electrospray ionization (ESI) modes. For positive mode, ESI source parameters were set as follows: Curtain Gas, 35 psi; Collision Gas, Medium; IonSpray Voltage, 5500 V; Source Temperature, 550 °C; Ion Source Gas 1, 60; and Ion Source Gas 2, 60. In negative mode, all parameters were identical except for the IonSpray Voltage, which was set to −4500 V.

Metabolite detection was performed in Multiple Reaction Monitoring (MRM) mode based on Novogene’s self-built database, novoDB. Qualitative identification of metabolites was achieved by matching precursor ions (Q1), product ions (Q3), retention time (RT), declustering potential (DP), and collision energy (CE) with reference standards in the database. Relative quantification of metabolites was determined based on the integrated chromatographic peak areas of Q3 quantitative product ions. Peak extraction, integration, and baseline correction were processed using SCIEX OS software (Version 1.4), with the main parameters configured as follows: minimum peak height, 500; signal-to-noise ratio (S/N), 5; and Gaussian smooth width, 1.

Following the acquisition of relative quantitative data, statistical analyses were conducted in the R environment (version 4.6.0). Principal Component Analysis (PCA) was performed to evaluate global metabolic variation among samples and intra-group reproducibility. Differentially accumulated metabolites (DAMs) were identified based on the criteria of Variable Importance in Projection (VIP) ≥ 1.0, |log_2_FC| ≥ 1, and Welch’s *t*-test *p*-value < 0.05. Volcano plots were generated using the ggplot2 package (RStudio version 2026.05.0, Posit Software, PBC, Boston, MA, USA) to visualize the magnitude of fold changes and statistical significance of metabolites between comparison groups.

### 2.5. Gene Co-Expression Network Analysis and WGCNA Validation

Gene co-expression networks were constructed based on transcript abundance data log_2_(FPKM + 1) derived from RNA-seq analysis. Pairwise Pearson correlation coefficients (PCCs) were calculated across all expression profiles using the cor function in R. Genes exhibiting strong co-expression relationships (PCC > 0.85) with at least one anthocyanin structural or regulatory gene were defined as core co-expressed genes, while those with extremely high correlations (PCC > 0.97) with key structural genes were classified as highly associated genes. Only gene pairs with PCC > 0.97 were retained for final network construction. Network visualization was performed in Cytoscape v3.10.0 [48] using an edge-weighted force-directed layout. In this representation, edge lengths reflect correlation strength, with shorter edges denoting more robust co-expression relationships.

To validate the accuracy and reliability of these co-expression relationships at the global transcriptome level, Weighted Gene Co-expression Network Analysis (WGCNA) was further performed. Expression data were preprocessed through multi-step filtering: genes with expression levels < 1 across all samples were removed; genes with a median absolute deviation (MAD) greater than the first quartile (Q1) were retained to exclude the 25% least variable genes; and genes with a coefficient of variation (CV > 0.5) were selected. Ultimately, 16,462 highly variable genes were retained for network construction using the WGCNA package in R. A signed weighted co-expression network was generated, and a soft-thresholding power of β = 9 was selected based on the scale-free topology fit index (R2 > 0.85). The adjacency matrix was transformed into a topological overlap matrix (TOM), and hierarchical clustering was performed using the average linkage method based on 1−TOM as the dissimilarity metric. Modules were identified using the cutreeDynamic function (deepSplit = 2, minimum module size of 50 genes), and modules with module eigengene correlations exceeding 0.8 were merged.

## 3. Results

### 3.1. Morphological, Anatomical, and Micromorphological Floral Characteristics of R. coccinea

**Figure 1:**
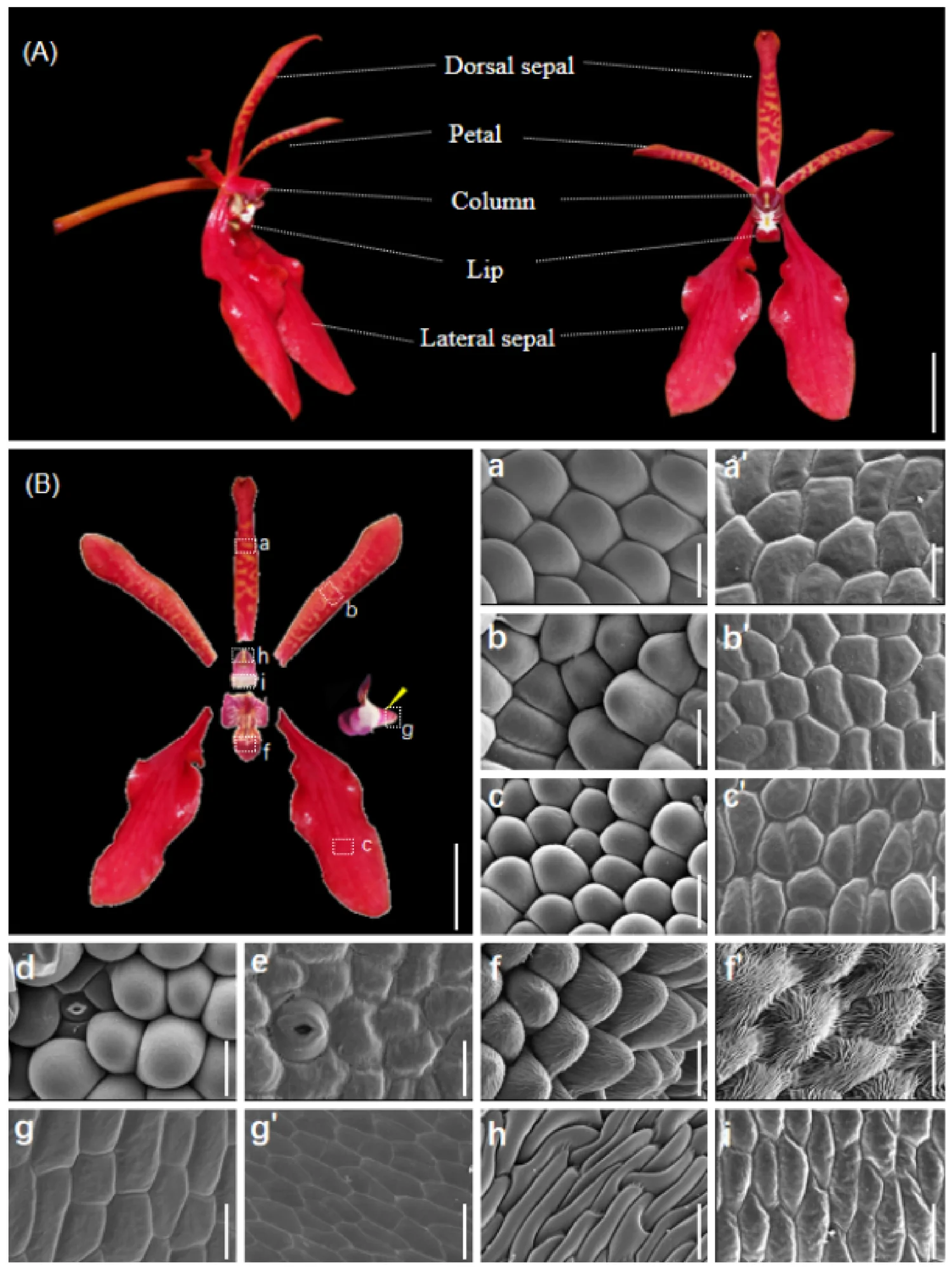
Floral morphology and epidermal cell specialization in *R. coccinea*. (**A**) Top and lateral views of mature flowers; (**B**) Dissected mature floral organs and representative scanning electron microscopy images showing epidermal cell morphology across floral tissues; (**a**–**c**), Conical epidermal on the adaxial surfaces of dorsal sepal, lateral sepal and petal, respectively; (**a′**–**c′**), Pavement epidermal cells on the abaxial surfaces of dorsal sepal, lateral sepal and petal, respectively; (**d**,**e**), Representative stomata distributed on the adaxial and abaxial surfaces of sepals and petals; (**f**), Conical epidermal cell with surface ornamentation on the adaxial surface of lip; (**f′**), Striated epidermal cells with surface ornamentation on the abaxial surface of lip; (**g**), Sub-rectangular interlocking epidermal cells on the adaxial surface of the spur; (**g′**), Flattened pavement cells on the abaxial surface of the spur; (**h**), Elongated/rod-shaped cells at the distal region of column; (**i**), Elongated interlocking epidermal cells at the basal region of column. Yellow arrow indicates the spur of the lip. Scale bars: flower image (**A**,**B**) 1 cm; SEM images (**a**–**i**) 50 µm.

To gain insights into the floral morphology and structural organization of the Flame Orchid, we investigated *R. coccinea*, a representative wild species distributed on Hainan Island. The flower is zygomorphic, with a single oblong-narrowly elliptic dorsal sepal that is red with red-blotched yellow. The lateral sepals are ovate, widening from a linear base; their margins are undulate, bright red, spreading vertically downwards, and slightly divergent or overlapping. The petals are linear and similar to the dorsal sepal. The lip is small and shallowly three-lobed. The side lobes feature red stripes, while the mid-lobe is yellowish-white with red stripes and a red surface, ending in an acute apex. Both the column and anther cap are red, with red bands at the column’s base and a distinct yellow band across the middle of the anther cap (Figure 1A). Clearly, sepal, petal, and lip differ markedly in morphology, structure, and color.

To clarify the differences in epidermal cell morphology among these floral organs, we examined their epidermal cells using scanning electron microscopy (SEM). We found that the adaxial epidermal cells of the sepals and petals were uniformly conical, whereas their abaxial epidermal cells were consistently pavement-like, with stomata occasionally scattered across both structures (Figure 1B(a–e)). The lip exhibited conical epidermal cells with surface modifications on the adaxial side and striated epidermal cells with surface modifications on the abaxial side (Figure 1B(f,f′)). By contrast, the spur of the lip possessed sub-rectangular interlocked epidermal cells on its adaxial surface and flattened pavement cells on its abaxial surface (Figure 1B(g,g′)). Furthermore, the column displayed elongated rod-shaped cells at the apex and elongated interlocked epidermal cells at the base (Figure 1B(h,i)). These observations suggest that the identity specification programs of distinct floral organs, including the dorsal sepal, petals, lateral sepals, the lip, and the gynostemium, may differ substantially.

### 3.2. Evolution and Expression of Floral Organ Identity Genes in R. coccinea

To gain more insights into the molecular mechanisms underlying the development and evolution of Flame Orchids, we first retrieved putative orthologs of floral organ identity genes from the de novo assembled reference transcriptome of *R. coccinea*, generated from pooled RNAs of floral buds, leaves, stems, and roots at different developmental stages. This reference transcriptome contained 39,390 predicted protein-coding genes. Phylogenetic analyses of floral organ identity genes and floral symmetry genes revealed that, while *AGL6-1*, *AGL6-2*, *AP3-1*, *AP3-2*, and *PI* lineage genes are predominantly maintained as single-copy orthologs within their respective sub-family clades, the *AG*, *SEP*, *DIV*, and *RAD* lineages exhibit lineage-specific expansions or increased copy numbers in certain taxa, such as *Renanthera*. For the *AG* and *SEP* lineages, the transcriptome-based phylogenetic results suggested possible lineage-specific duplication, giving rise to the *AG1*, *AG2*, and *AG3* clades and the *SEP1*, *SEP2*, and *SEP3* clades (Supplemental Figures S1 and S2; Supplemental Data Set S1). Among the floral symmetry genes, the two previously identified *Orchis CYC* lineages underwent duplication, resulting in the *CYC1* and *CYC2* clades. In some genera, a subsequent duplication of *CYC2* gave rise to *CYC3*. This evolutionary trend is mirrored in *Renanthera*, which features three copies, namely *CYC1*, *CYC2*, and *CYC3*. Similar to the evolutionary history of *CYC* genes, the *DIV* gene also produced three copies: *DIV1*, *DIV2*, and *DIV3* in *Renanthera* (Supplemental Figure S3; Supplemental Data Set S1).

**Figure 2:**
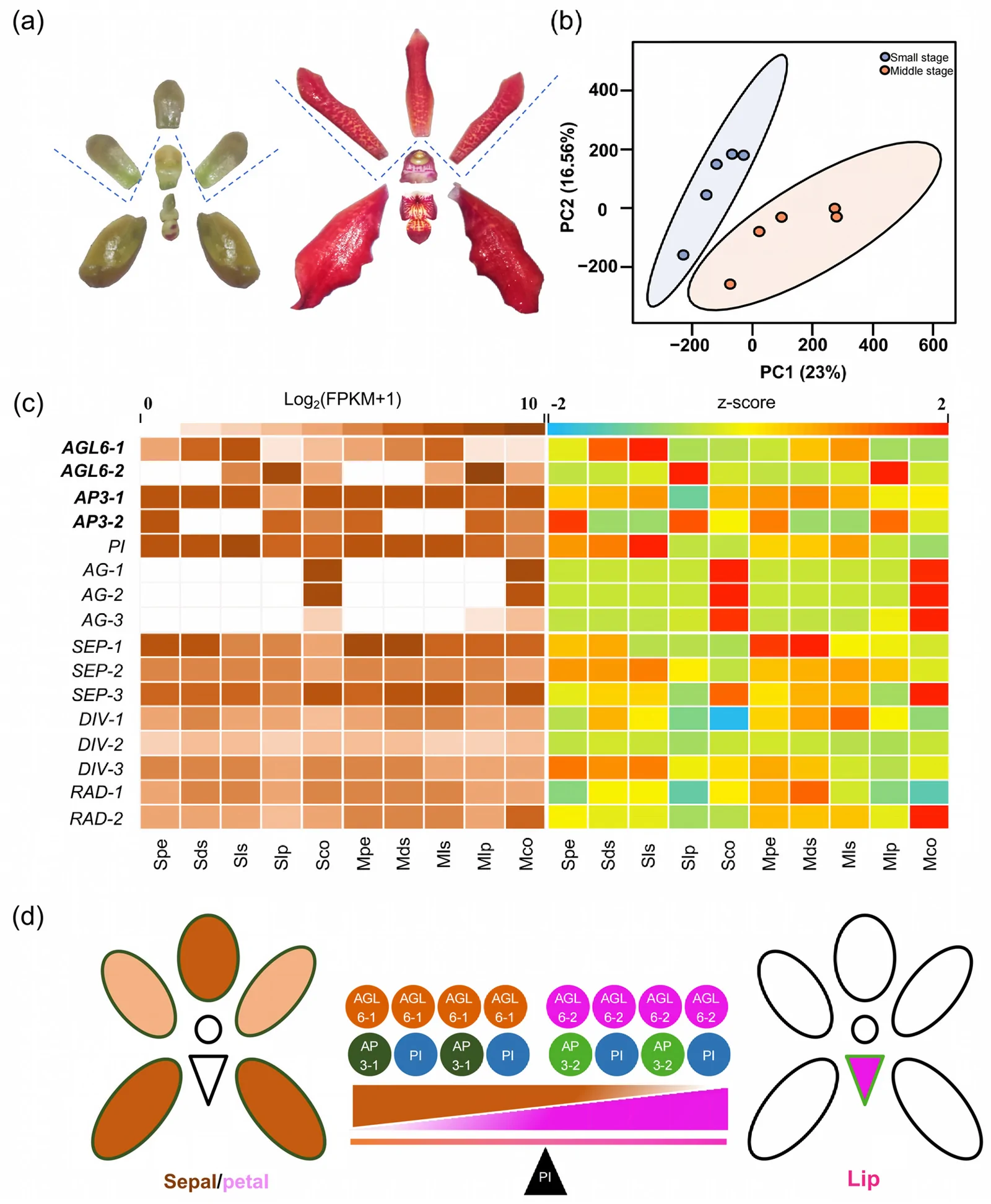
Expression landscape of floral organ identity and symmetry-related genes reveals the perianth development model of *R. coccinea*. (**a**) Morphology of dissected floral organs from small (left) and middle (right) stage floral buds. (**b**) Principal component analysis of the 10 floral organ transcriptomes from small and middle stage floral buds. (**c**) Spatiotemporal expression profiles of floral organ identity genes and floral symmetry-related genes across floral tissues and developmental stages. The left heatmap represents log_2_ (FPKM+1)-transformed expression values, whereas the right heatmap represents Z-score-normalized expression values. Genes showing prominent expression differences in the perianth are highlighted in bold. (**d**) Proposed regulatory model for perianth identity specification in *R. coccinea*. PI may provide a basal perianth identity program, whereas AP3-1/AGL6-1 are associated with sepal/petal identity, and AP3-2/AGL6-2 are associated with lip identity. Sco, column of small floral bud; Spe, petal of small floral bud; Sds, dorsal sepal of small floral bud; Sls, lateral sepal of small floral bud; Slp, lip of small floral bud; Mco, column of middle floral bud; Mpe, petal of middle floral bud; Mds, dorsal sepal of middle floral bud; Mls, lateral sepal of middle floral bud; Mlp, lip of middle floral bud (*n* = 3 independent biological replicates).

To investigate the expression patterns of the aforementioned genes, transcriptome profiling was performed among dorsal sepal/petals, lateral sepals, the lip, and the column from *R. coccinea* at both the small (marking the completion of organ differentiation and the onset of coloration) and middle (representing the full maturation of floral pigmentation) stages (Figure 2a). Principal Component Analysis (PCA) revealed significant stage-specific clustering, with PC1 and PC2 accounting for 23% and 16.56% of the total variance, respectively (Figure 2b; Supplemental Figure S4; Supplemental Data Set S2). These results indicate that the developmental stage is the primary driver of transcriptomic variation, despite the inclusion of various organ types, while also indicating clear biological structure and overall reproducibility among the RNA-seq samples. Digital gene expression profiles revealed clear expression divergence of *AP3* and *AGL6* paralogs across floral organs (Figure 2c; Supplemental Data Set S3). *AP3-1* was predominantly expressed in the dorsal sepal/petals and lateral sepals, whereas *AP3-2* showed a preference for the petals and lip. Similarly, *AGL6-1* exhibited high expression levels in both dorsal and lateral perianth organs, displaying a progressively increasing trend along the floral axis of symmetry. In contrast, *AGL6-2* was primarily restricted to the lip, with its expression intensity showing a decreasing gradient along the same axis. These results are consistent with findings in *Oncidium* species [25], at least at the small and middle floral developmental stages. *AG-1*, *AG-2*, and *AG-3* were specifically expressed in the column, with *AG-1* and *AG-2* showing significantly higher expression levels in this structure. While *SEP-1* and *SEP-3* exhibited higher expression levels among the three *SEP* genes, these genes were found to be expressed across all types of floral organs. Interestingly, although *DIV* and *RAD* were also widely expressed across various floral organs, their transcript levels were markedly lower than those of floral organ identity genes (Figure 2c; Supplemental Data Set S3). Furthermore, no detectable expression of *CYC* genes was observed at these two developmental stages (Supplemental Data Set S3), possibly because the peak expression of these early-acting symmetry genes occurs prior to the developmental stages sampled in this study. To validate the reliability of the transcriptomic profiling data, the expression patterns of four selected genes (*AGL6-1*, *AGL6-2*, *AP3-1*, and *AP3-2*) were examined using qRT-PCR. The qRT-PCR results were highly consistent with the RNA-seq expression trends, further confirming the robustness and accuracy of our transcriptomic dataset (Supplemental Figure S10).

Taken together, these findings suggest that duplicated and diversified *AP3-* and *AGL6*-lineage genes represent strong candidates underlying the origin of the complex floral morphology in Flame Orchids, further supporting that the P-code hypothesis is applicable to perianth formation in this group (Figure 2d).

### 3.3. Genes Specifically and Preferentially Expressed in the Lip of R. coccinea

**Figure 3:**
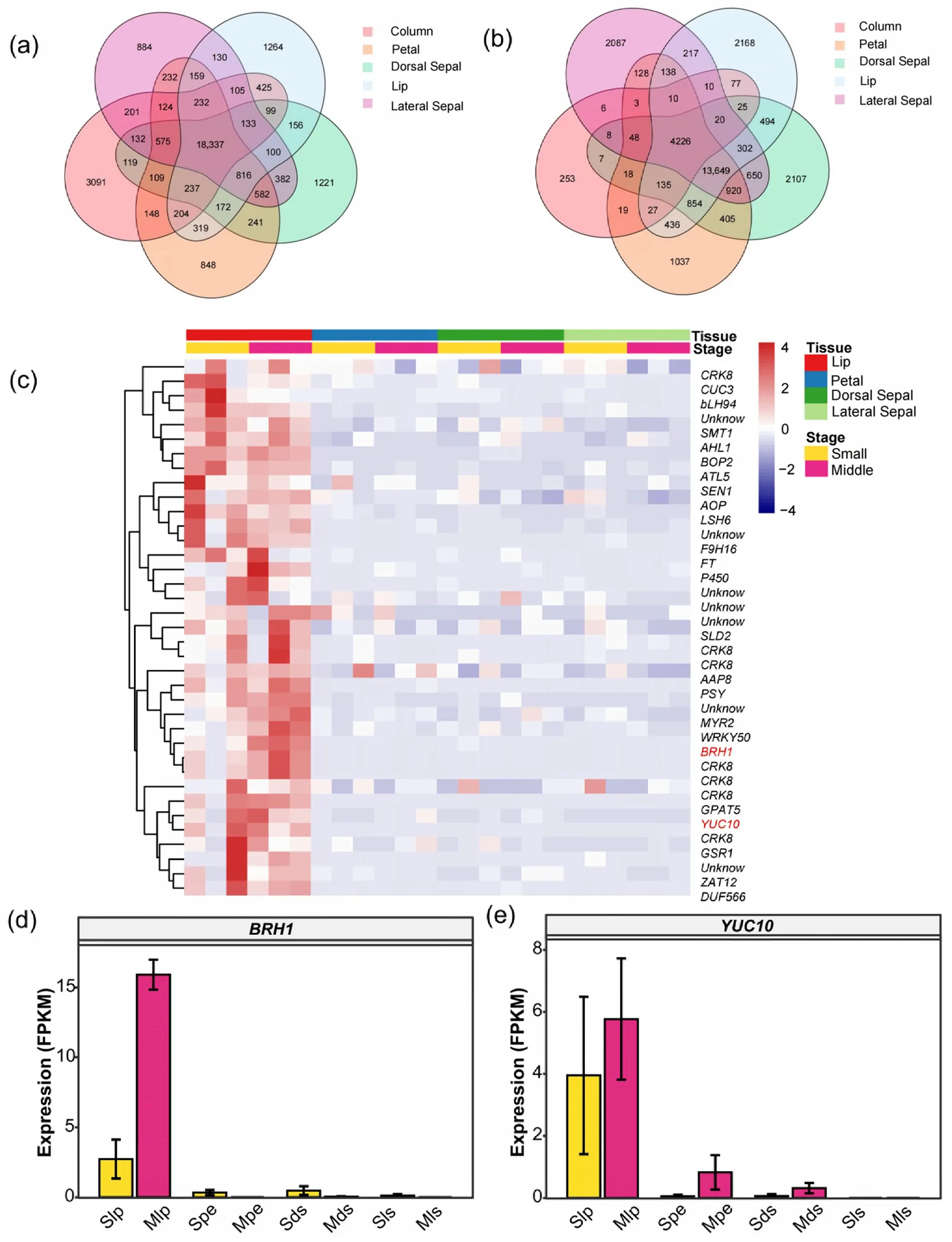
Identification of genes preferentially expressed in various floral organs, specifically highly expressed in the lip, and associated with specialization of the lip, particularly the development of the spur in *R. coccinea*. (**a**,**b**) Venn diagrams showing the numbers of genes preferentially expressed in different organs in small stage (**a**) and middle stage (**b**) floral buds. (**c**) Heatmap showing the top 37 genes specifically and highly expressed in the lip of *R. coccinea*. (**d**,**e**) Expression profile of two candidate spur development-associated genes, *BRH1* (**d**) and *YUC10* (**e**), across floral organs. Sco, column of small floral bud; Spe, petal of small floral bud; Sds, dorsal sepal of small floral bud; Sls, lateral sepal of small floral bud; Slp, lip of small floral bud; Mco, column of middle floral bud; Mpe, petal of middle floral bud; Mds, dorsal sepal of middle floral bud; Mls, lateral sepal of middle floral bud; Mlp, lip of middle floral bud (*n* = 3 independent biological replicates).

To understand the uniqueness of the lip, we comprehensively compared the gene expression profiles across five distinct floral organs (column, petal, dorsal sepal, lateral sepal, and lip) at both the small (Figure 3a; Supplemental Data Set S4) and middle (Figure 3b; Supplemental Data Set S4) floral bud stages. Of all the genes expressed, 1264 and 2168 were identified as lip-specific (defined as FPKM ≥ 1.0 in the lip and <1.0 in all other floral organs) at the small and middle stages, respectively. For comparison, the numbers of organ-specific genes at the small and middle stages were 848 and 1037 in the petal, 1221 and 2107 in the dorsal sepal, 884 and 2087 in the lateral sepal and 3091 and 253 in the column, respectively. Given that the orchid lip is typically a highly specialized organ, our study further focused on the lip tissue to identify genes related to its development and morphological elaboration. By screening for genes that maintained lip-specific expression across both developmental stages (FPKM ≥ 1.0), we identified a core set of 37 genes (Supplemental Data Set S5). Among these, two genes brassinosteroid-responsive RING H2 (*BRH1*) and flavin-containing monooxygenase *YUCCA10* (*YUC10*) are closely associated with cell elongation and hormone responses (Figure 3c).

Quantitative gene expression analysis (FPKM) supported the lip-enriched expression patterns of these two candidate genes (Figure 3d,e). The results showed that *BRH1* and *YUC10* were transcribed at high abundance almost exclusively in the lip, remaining virtually silent in the other floral organs. Notably, *BRH1* exhibited a highly significant stage-dependent expression pattern (Figure 3d). Its expression was dramatically upregulated in the middle lip, far exceeding the levels observed at the small stage. This pattern suggests that *BRH1* may participate in the later stages of labellum specialization, particularly in spur elongation and morphogenesis. Concurrently, *YUC10*, which encodes a key enzyme involved in local auxin biosynthesis, maintained high and specific expression in the lip at both developmental stages (Figure 3e). This expression pattern suggests that localized auxin biosynthesis may contribute to lip formation and development in *R. coccinea*, although functional validation is required to confirm this role.

### 3.4. Spatio-Temporal Accumulation of Cyanidin Derivatives Drives Floral Color Divergence Between Dorsal and Lateral Sepals in R. coccinea

**Figure 4:**
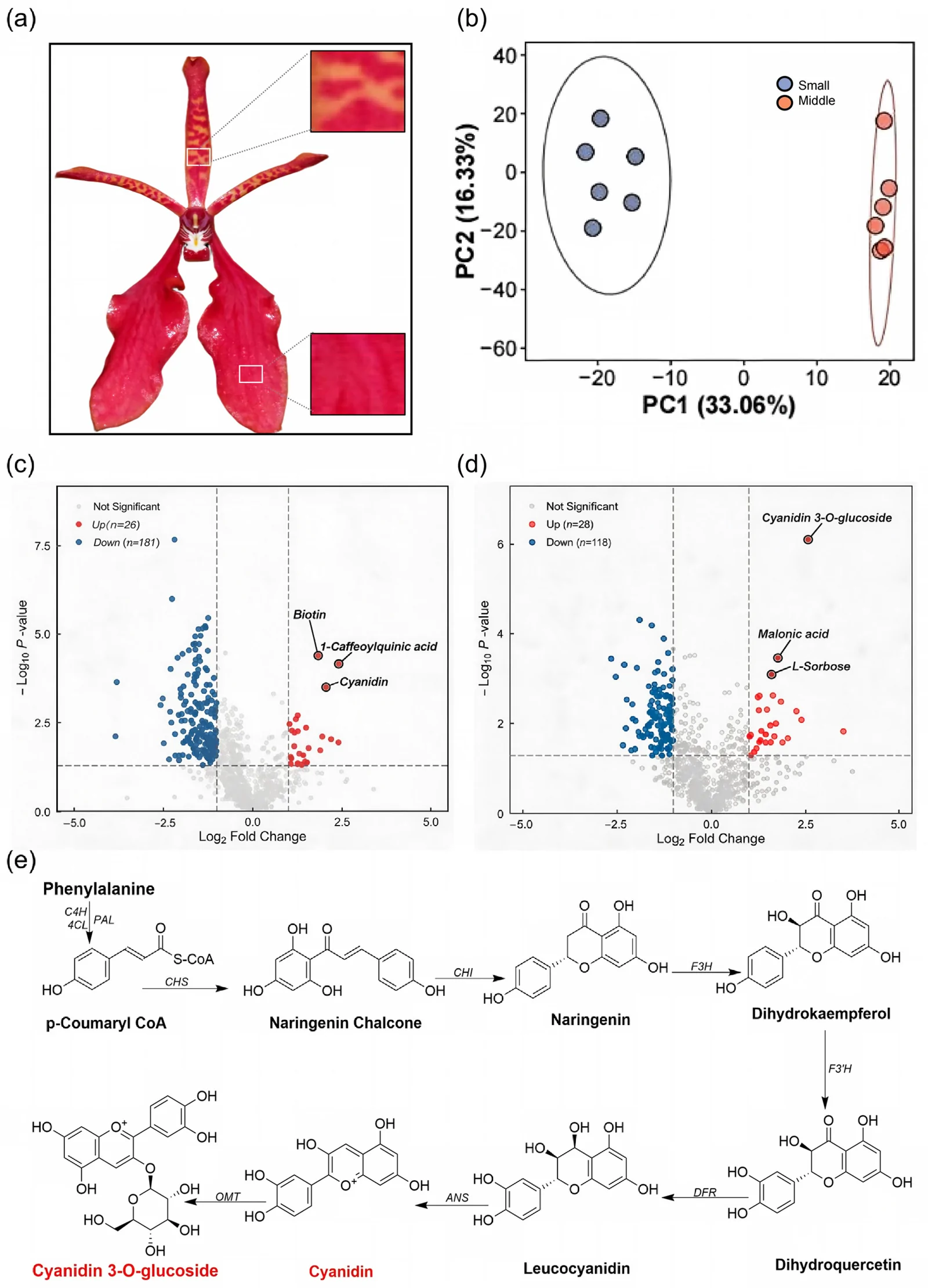
Identification of the floral color pigments and metabolic network in *R. coccinea*. (**a**) Floral morphology and pigmentation patterns of mature floral tissues. Boxed regions highlight the pigmentation differences between the dorsal and lateral sepal. (**b**) Principal component analysis of the 12 metabolome profiles (4 groups, 3 biological replicates per group) from dorsal and lateral sepal at small and middle developmental stages. Each point represents an individual biological replicate. (**c**,**d**), Volcano plots showing differentially accumulated metabolites between small and middle stages in the dorsal sepal (**c**) and lateral sepal (**d**). (**e**) Cyanidin 3-O-glucoside biosynthesis pathway in *R. coccinea* (*n* = 3 independent biological replicates).

To elucidate the differences in the main chromogenic substances between the red-blotched yellow dorsal sepals and petals and the pure red lateral sepals of *R. coccinea* (Figure 4a), we performed widely targeted metabolomic profiling on both tissue types at the small and middle developmental stages (Supplemental Data Set S6). Principal component analysis (PCA) revealed a distinct and significant separation between the two stages along the PC1 axis (33.06%; Figure 4b), indicating a profound spatiotemporal evolution of metabolic profiles during floral maturation. Furthermore, samples from the two tissue types (dorsal sepal vs. lateral sepals) formed distinct clusters along the PC2 axis (16.33%; Figure 4b), suggesting that tissue-specific metabolic differentiation also contributes substantially to the overall variance. Together, these results demonstrate that developmental stage and tissue type are two major determinants of metabolic variation, providing a solid basis for further identifying the specific metabolites responsible for the distinct color phenotypes.

A total of 207 differentially accumulated compounds were identified in the dorsal sepal across the two developmental stages. Analysis of differentially accumulated metabolites (DAMs) revealed 26 up-regulated and 181 down-regulated compounds (Supplemental Data Set S7). Among the 26 up-regulated metabolites, three key metabolites showed the markedly higher accumulation, notably cyanidin a key pigment most closely associated with floral coloration (Figure 4c; Supplemental Figures S8 and S9). Research indicates that cyanidin is inherently unstable under physiological conditions, typically requiring glycosylation for structural stability [49]. In *R. coccinea*, this inherent instability may be associated with localized pigment degradation, potentially contributing to the specific pigmentation patterns observed. Since yellow serves as the ground coloration, it is hypothesized that the breakdown of red cyanidin pigments could unmask the underlying yellow background, leading to the red-blotched phenotype where areas of cyanidin depletion reveal the yellow base while persisting pigments form red patches. However, as whole dorsal sepals were homogenized for this bulk metabolomic analysis, future studies using high-resolution spatial metabolomics (e.g., MALDI-MSI) or laser capture microdissection will be required to definitively confirm this spatial pigment localization model. In contrast, we identified a total of 146 differentially accumulated compounds in the lateral sepals across the two developmental stages and found that the lateral sepals showed marked accumulation of cyanidin 3-O-glucoside during development, with its fold-change significantly surpassing other metabolites (Figure 4d; Supplemental Figures S8 and S9). By integrating our findings within the framework of the known anthocyanin biosynthetic pathway (Figure 4e), these results suggest that quantitative and tissue-specific accumulation of cyanidin and its derivatives contributes to the distinct coloration patterns observed between the dorsal and lateral sepals of *R. coccinea*.

### 3.5. Characterization of the Anthocyanin Biosynthetic Pathway and Its Transcriptional Regulatory Network in R. coccinea

**Figure 5:**
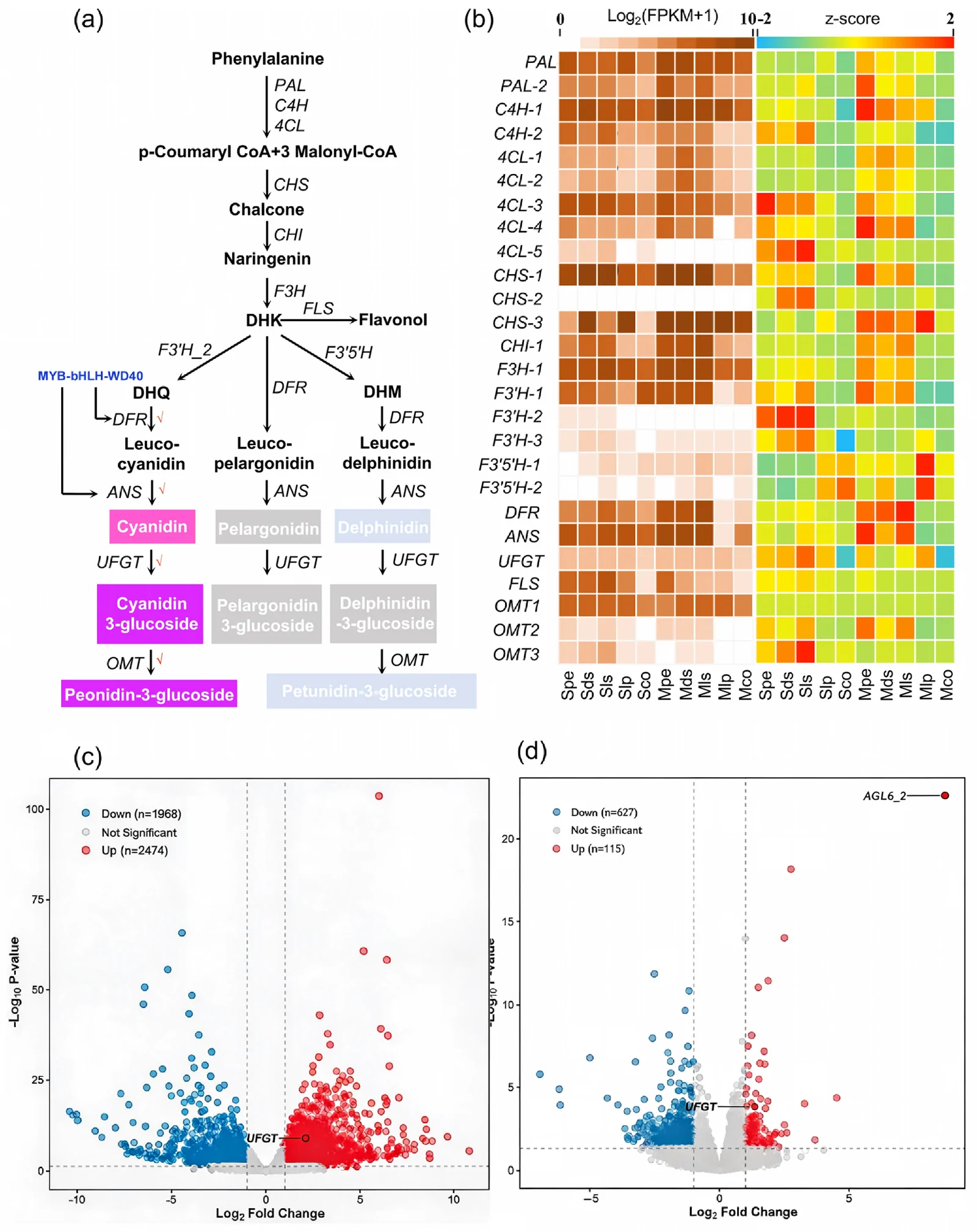
Transcriptomic analysis of the anthocyanin biosynthesis pathway and key differentially expressed genes associated with floral pigmentation in *R. coccinea*. (**a**) Schematic representation of the anthocyanin biosynthesis pathway and major structural enzymes involved in pigment formation. (**b**) Spatiotemporal expression profiles of anthocyanin biosynthesis-related genes across developmental stages (S, small stage; M, middle stage) and floral tissues (left panel: log_2_(FPKM+1) heatmap; right panel: Z-score normalized heatmap). (**c**) Volcano plot showing differentially expressed genes (DEGs) between small and middle stage lateral sepals. (**d**) Volcano plot showing differentially expressed genes between dorsal sepals and lateral sepals at the middle stage. The structural gene *UFGT* and transcription factor *AGL6-2* are highlighted in the volcano plots, providing molecular insights into anthocyanin accumulation and tissue-specific floral coloration in *R. coccinea* (*n* = 3 independent biological replicates).

To elucidate the molecular mechanisms underlying red pigmentation (cyanidin), we identified candidate genes involved in anthocyanin biosynthesis and examined their expression patterns. A total of 26 candidate structural genes were identified in the anthocyanin biosynthetic network (Figure 5a,b; Supplemental Data Set S8). Except for chalcone synthase (*CHS-2*) and flavonoid 3′,5′-hydroxylase (*F3′5′H*), which showed no detectable or low expression, all other genes exhibited high expression signals at two stages. As expected, consistent with the metabolomic data, the high expression of *F3′H* directs the anthocyanin biosynthetic flux primarily toward the cyanidin pathway (Figure 5a,b). In contrast, the extremely low expression of *F3′5′H* hinders delphinidin synthesis, while the coordinated expression of *F3′H* and dihydroflavonol 4-reductase (*DFR*) channels metabolic flux predominantly toward the cyanidin branch, thereby limiting pelargonidin production. Collectively, these molecular mechanisms account for why cyanidin is the predominant pigment responsible for the coloration of Flame Orchid. Additionally, a significant down-regulation of *F3′H*, *DFR*, and anthocyanidin synthase (*ANS*) was observed in the lip and column as development progressed toward the middle stage. This reduction likely limits the intensity of red pigmentation, providing a molecular basis for why these tissues do not achieve a completely deep red hue.

To elucidate the molecular basis of the striking dorsal/lateral floral color divergence observed in the Flame Orchid, a pattern uniquely characterized by the accumulation of cyanidin in dorsal tissues versus cyanidin 3-O-glucoside in lateral sepals, we performed comprehensive transcriptomic and differential gene expression (DGE) profiling. Across the transition from the small development stage to middle (M) developmental stage, a total of 4442 DEGs (2474 up-regulated and 1968 down-regulated) were identified in lateral sepals, whereas only 742 DEGs (115 up-regulated and 627 down-regulated) were detected in dorsal sepals. Based on the metabolic profile, UDP-glucose: flavonoid 3-O-glucosyltransferase (UFGT), which mediates the biochemical conversion of cyanidin into its stable glucoside form, emerged as the primary candidate enzyme in this pathway. To verify this hypothesis, we first conducted a temporal comparative analysis of lateral sepals across the two stages (Supplemental Data Set S9). This analysis revealed that *UFGT* was significantly upregulated as the flowers transitioned from the small to the middle stage (Figure 5c; Supplemental Figure S10). To test whether *UFGT* expression correlates with spatial color patterning, we compared transcript levels between dorsal and lateral sepals during the middle stage (Supplemental Data Set S10). The results showed that *UFGT* was expressed at significantly higher levels in lateral sepals than in dorsal sepals, closely aligning with the localized accumulation of cyanidin glucosides (Figure 5d). While these expression correlations do not directly establish causality without functional validation, they identify *UFGT* as a primary candidate gene associated with dorsal/lateral pigmentation divergence, providing a solid lead for future functional studies.

### 3.6. Identification of Candidate Regulatory Genes Underlying Dorsal/Lateral Color Divergence via Co-Expression Network Analysis

**Figure 6:**
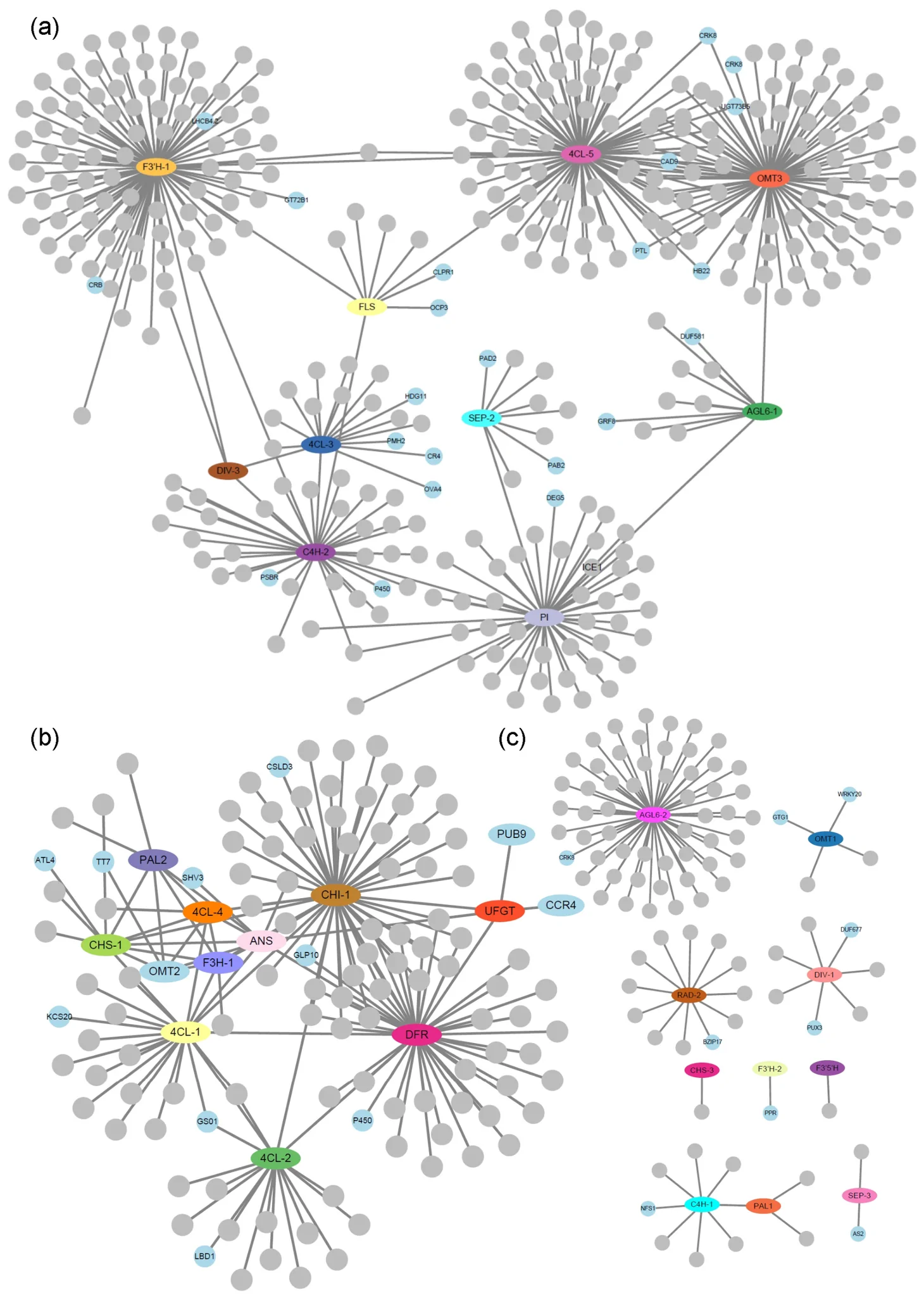
Co-expression network analysis of floral development and anthocyanin biosynthesis pathway genes. (**a**) Regulatory network of hub genes involved in both floral development and anthocyanin biosynthesis pathway genes. (**b**) Regulatory network consisting exclusively of hub genes from the anthocyanin biosynthesis pathway genes. (**c**) Regulatory networks/modules comprising only one or two hub genes from either floral development or anthocyanin biosynthesis pathway genes. Nodes represent genes, and edges indicate co-expression correlations. Key functional hub genes are color-coded. These networks further elucidate the complex interaction mechanisms between structural genes and co-expression genes in determining the specific floral coloration of *R. coccinea* (*n* = 3 independent biological replicates).

During our screening for genes underlying the color divergence between lateral and dorsal sepals, we observed that the floral developmental gene *AGL6-2* was significantly up-regulated in lateral sepals relative to dorsal sepals (Figure 5d). This finding led us to hypothesize that floral organ identity genes might coordinate with the anthocyanin biosynthetic pathway to jointly regulate spatial floral coloration. To test this hypothesis systematically, we integrated all identified floral developmental genes together with all core anthocyanin structural genes as anchors into a co-expression network analysis. Using stringent criteria, including PCC > 0.85 among anchor genes and PCC > 0.97 between each anchor gene and its associated co-expression genes, we identified co-expression partners for 33 genes (Supplemental Data Set S11). These genes were partitioned into distinct co-expression groups. One network connected anthocyanin structural genes flavonol synthase (*FLS*), flavonoid 3′-hydroxylase (*F3′H-1*), cinnamate-4-hydroxylase (*C4H-2*), 4-coumarate:CoA ligase (*4CL-3/5*) and o-methyltransferase (*OMT3*) with floral developmental genes (*AGL6-1*, *SEP4*, *PI*, and *DIV-3*), suggesting that these organ identity genes not only specify organ identity but may also coordinate floral pigmentation through interactions with anthocyanin biosynthetic genes (Figure 6a). A second network contained several anthocyanin structural genes, including phenylalanine ammonia-lyase (*PAL-2*), *4CL-1/2/4*, *CHS-1*, chalcone isomerase (*CHI-1*), flavanone 3-hydroxylase (*F3H-1*), *DFR*, *ANS*, *UFGT*, and *OMT2*, indicating that *UFGT*, which was identified in our differential expression analysis of dorsal and lateral sepals, is tightly co-regulated alongside core structural genes in the anthocyanin biosynthetic pathway. Co-expression analysis revealed that transparent testa 7 (*TT7*), *F3′H* and one P450 family gene exhibited strong co-expression patterns with *CHS-1* and *DFR*, respectively. These enzymatic components may contribute to anthocyanin biosynthetic flux in the perianth of the Flame Orchid (Figure 6b). Additionally, pairwise co-expression was observed for *PAL-1/C4H-1* and *SEP2/3* (Figure 6c), whereas the remaining genes failed to incorporate into any co-expression modules; this suggests that these organ identity genes may not play a primary role in the regulation of floral pigmentation in this context. These results further demonstrate that *UFGT* is indeed the key gene driving the differentiation of coloration patterns between the dorsal and lateral perianths in *R. coccinea*. Intriguingly, while traditional transcription factors (TFs) were absent from the *UFGT*-containing module, two non-canonical regulatory genes, plant U-box protein 9 (*PUB9*, an E3 ubiquitin ligase) and carbon catabolite repressor 4 (*CCR4*, a key component of the deadenylase complex), were found to be tightly co-expressed with *UFGT*. To further validate the robustness of our findings, we performed Weighted Gene Co-expression Network Analysis (WGCNA). The module analysis from WGCNA was highly consistent with our initial co-expression network results, reinforcing the reliability of our data (Supplemental Figures S6 and S7). This suggests that the distinct coloration of lateral sepals may be governed by post-translational or post-transcriptional mechanisms rather than direct transcriptional activation alone.

## 4. Discussion

### 4.1. Conservation of Floral Organ Identity Mechanisms and Regulatory Model Construction in Flame Orchid

In this study, we conducted the first systematic identification of floral organ identity and zygomorphy-related genes in the representative species of *R. coccinea*, and characterized their expression profiles during key developmental stages (small and middle buds). Among the 11 identified organ identity genes, all except *AG-3* exhibited high expression abundance, underscoring their pivotal roles in the floral ontogeny of Flame Orchid. Our results revealed that *AGL6-1*, *AP3-1*, and *AP3-2* were predominantly expressed in the dorsal floral organs, whereas *AGL6-2* and *AP3-2* were specifically and significantly upregulated in the lip. Class C *AG* genes were strictly confined to the column. This spatial expression pattern is highly congruent with those observed in typical orchids such as *Oncidium* and *Phalaenopsis* [25,50]. Based on the conservation of these expression patterns and functional insights from closely related species, such as the non-functional expression of *AP3-2* in the petals of *Oncidium* [25] we propose that Flame Orchid follows an Orchid Code-like regulatory pattern. Specifically, dorsal organ identity is primarily defined by *AGL6-1* and *AP3-1*, while lip identity is co-determined by *AGL6-2* and *AP3-2*, and column identity is maintained by *AG* genes. Accordingly, we have constructed a genetic regulatory model for floral development in Flame Orchid. These findings not only refine the molecular framework of the genus *Renanthera* but also further substantiate the high degree of conservation in floral organ identity mechanisms across the Orchidaceae at the family level.

### 4.2. AP3-like and AGL6-like Genes May Contribute to Floral Zygomorphy in Flame Orchid

In this study, eight genes associated with floral zygomorphy were identified. Notably, the *CYC-like* genes, which serve as the traditional core regulators of symmetry, were nearly undetectable across the two sampled developmental stages. This absence may be attributed to highly restricted spatio-temporal expression, potentially initiating at an extremely early stage, or it may suggest that *CYC* genes are not the primary factors maintaining zygomorphy in Flame Orchid. Furthermore, other classes of symmetry-related genes (i.e., *DIV* and *RAD*) generally exhibited lower expression levels compared to MADS-box genes and showed broad, non-tissue-specific expression patterns. These observations are congruent with findings in other orchid species, suggesting that the unique zygomorphic structures of Orchidaceae, particularly lip specialization, may not be predominantly governed by the traditional *CYC* pathway. Instead, this process appears to be driven by the ectopic expression or functional divergence of organ identity genes (MADS-box). Evolutionary evidence further supports this hypothesis: in the basal orchid genus *Apostasia*, the absence of specific *AP3* subfamilies fails lip specialization, leading to actinomorphic (radially symmetrical) features similar to those of the petals [48]. Moreover, in *Cymbidium sinense*, the development of floral symmetry has been linked to the expression patterns of *Cymbidium sinense APETALA3-2* (*CsAP3-2*) and *Cymbidium sinense AGAMOUS-LIKE 6-2* (*CsAGL6-2*) [30]. Integrating our data, we propose that the pivotal role of *AP3*-class and *AGL6*-class genes in lip specialization not only defines organ identity but also serves as the core genetic driver for the evolution of floral zygomorphy in Flame Orchid.

### 4.3. Molecular Mechanisms Underlying Lip Development in Flame Orchid

Beyond the core floral organ identity genes, tissue-specific expression analysis identified two hormone-associated, lip-enriched genes, *YUC10*, encoding an auxin biosynthesis-related flavin-containing monooxygenase, and *BRH1*, encoding a brassinosteroid-responsive RING-H2 protein. Both genes showed strong lip-enriched expression in Flame Orchid. Given that *AP3*-class genes have been shown to contribute to lip identity in orchids [51]. We propose that these genes may act as downstream components of hormone-associated pathways involved in lip morphogenesis. The spur represents the most conspicuous specialized feature of the Flame Orchid lip. The spur is a critical morphological trait for pollinator adaptation in Orchidaceae, and its development is governed by a complex hormonal landscape. In the model plant *Aquilegia*, studies have shown that the auxin response factors (*ARF6/8*) maintain high expression levels during spur development, determining spur length by regulating longitudinal cell elongation rather than cell division; conversely, inhibition of their function results in significantly shortened spurs due to restricted cell elongation [52]. In addition to auxin, the brassinosteroid (BR) signaling pathway is equally pivotal. Exogenous BR application significantly increases spur length in *Aquilegia*, while the suppression of BR signaling components BES1/BZR1-Homologs (*BEH1/BEH3*) reduces cell-growth anisotropy, leading to spur stunting [25]. Furthermore, in *A. brevistyla*, the bHLH transcription factor increased Leaf Inclination Binding bHLH1-Like (*IBL1*) has been shown to control the curved growth of the spur by fine-tuning hormonal balance [53].

This hormone-driven mechanism of spur development exhibits functional convergence across diverse species. For instance, in *Impatiens*, genes encoding extensin proteins involved in cell-wall synthesis are specifically expressed in the spur, where they determine morphology by modulating anisotropic cell growth. Similarly, the auxin-binding protein ABP precisely regulates spur curvature by influencing differential cell division rates between the proximal and distal regions [54]. Integrating these findings with our results, the lip-specific high expression of the auxin biosynthesis gene *YUC10* and the BR-responsive gene *BRH1* suggests a similar regulatory logic: following the establishment of lip identity by MADS-box genes, the activation of hormonal signaling pathways is likely mediated by factors such as *YUC10* and *BRH1*, thereby inducing localized polar elongation or differential cell division. Such coordinated hormonal and cellular processes may contribute to the morphogenesis of complex lip structures, including the spur, in Flame Orchid. However, whether *YUC10* and *BRH1* indeed execute these regulatory functions remains to be experimentally confirmed through future functional genetic studies in *R. coccinea* and closely related orchid species.

### 4.4. Differential Expression of Structural Genes Is Associated with Dorsal/Lateral Floral Color Variation in Flame Orchid

Cyanidin has been reported as a major contributor to flower coloration in orchid plants [44,55]. In this study, we identified cyanidin aglycones and cyanidin-3-O-glucoside as the major pigments in the dorsal sepal and lateral sepals of Flame Orchid, respectively. Through developmental stage-specific analysis, we found that *UFGT* was differentially expressed between these organs (highly upregulated in the lateral sepals). This indicates that the two pigmentation patterns are associated with differential modification of anthocyanins, particularly through variation in *UFGT* expression. Such transcriptional divergence serves as the molecular foundation for the color variation between the dorsal and lateral floral organs of Flame Orchid. Indeed, direct transcriptional regulation of *UFGT* has been recognized as a pivotal mechanism governing anthocyanin accumulation and flower color dynamics in plants. For instance, recent evidence in lilac (*Syringa oblata*) showed that the SoNAC72-SoMYB44/SobHLH130 complex directly binds to and activates the *SoUFGT1* promoter to regulate anthocyanin biosynthesis during flower color fading [56]. Our findings further support the critical role of *UFGT* transcriptional regulation in driving tissue-specific anthocyanin glycosylation and floral color patterning.

Although anthocyanin biosynthesis is regulated by the MYB-bHLH-WDR (MBW) trimeric complex, recent studies have highlighted the critical roles of epigenetic and post-translational modifications (PTMs) in fine-tuning this pathway during plant development and environmental adaptation. Intriguingly, our analysis identified *PUB9* and *CCR4* as candidate genes strongly associated with *UFGT*. *PUB9* is a member of the E3 ubiquitin ligase family, and *CCR4* serves as the core catalytic component of the CCR4-NOT complex involved in mRNA degradation in eukaryotes. Research has demonstrated that both the E3-mediated degradation of transcription factors and the inhibition of MBW complex formation by R3-MYB factors are essential for maintaining anthocyanin homeostasis in plants [45,57]. This suggests that the spatial regulation of *UFGT* in Flame Orchid may be primarily governed by post-translational or post-transcriptional mechanisms. Specifically, the positive co-expression of *PUB9* with *UFGT* raises the possibility that ubiquitin-mediated protein turnover may influence anthocyanin modification, the patterns observed in Flame Orchid point toward a mode of action involving the targeted degradation of transcriptional repressors by *PUB9*. Furthermore, the co-expression of *CCR4* with *UFGT* highlights the significance of mRNA stability in floral pigmentation. The CCR4-NOT complex may also influence transcript stability of *UFGT* or its upstream regulators in the lateral sepals, thereby facilitating rapid and robust pigment accumulation.

## 5. Conclusions

In conclusion, this study provides a comprehensive multi-omics framework for understanding the molecular mechanisms governing floral organ identity, lip morphogenesis, and spatial color patterning in *R. coccinea*. Our results suggest that floral organ identity and zygomorphy follow an Orchid Code-like model associated with organ-specific expression of *AGL6* and *AP3* lineage genes, rather than traditional *CYC* pathways. Lip specialization and spur formation may be fine-tuned downstream by lip-enriched hormonal regulators (*YUC10* and *BRH1*). Furthermore, the distinct color divergence between dorsal and lateral sepals is fundamentally associated with differential anthocyanin glycosylation, where *UFGT* serves as a key candidate enzymatic driver. The strong co-expression of *UFGT* with *PUB9* and *CCR4* highlights the potential involvement of post-translational and post-transcriptional fine-tuning in pigmentation. While direct functional genetic validation will be required in future studies, these findings reveal key regulatory networks of orchid flower development and provide valuable candidate genes for molecular breeding in *Renanthera*.

## Data Availability

The RNA-seq datasets generated in this study have been deposited in the NCBI Sequence Read Archive (SRA) under accession number PRJNA1457784 and in the Gene Expression Omnibus (GEO) under accession number GSE330382. All other data supporting the findings of this study are included in this published article and its Supplementary Information files or are available from the corresponding author upon reasonable request.

## Supplementary Materials

The following supporting information can be downloaded at: https://www.mdpi.com/article/10.3390/biology15171497/s1, Supplemental Figure S1. Phylogenetic trees of other floral organ identity genes. Supplemental Figure S2. Phylogenetic trees of *SEP-like* genes. Supplemental Figure S3. Phylogenetic trees of floral symmetry genes. Supplemental Figure S4. Quality assessment and principal component analysis of transcriptomic data. Supplemental Figure S5. sample correlation analysis of transcriptomic data in *R. coccinea*. Supplemental Figure S6. Weighted gene co-expression network analysis (WGCNA) and expression profiles of key candidate modules. Supplemental Figure S7. Expression profiles of genes in WGCNA co-expression modules across different tissue samples. Supplemental Figure S8. Heatmap of 17 anthocyanin compounds in *R. coccinea*. Supplemental Figure S9. Bar chart of 17 anthocyanin compounds in *R. coccinea*. Supplemental Figure S10. Relative expression levels of key candidate genes determined by qRT-PCR in *R. coccinea*. Supplemental Data Set S1. Gene information used for phylogenetic analyses. Supplemental Data Set S2. Mean FPKM values of the 39,390 Genes across three replicates for each organ in *R. coccinea*. Supplemental Data Set S3. Mean FPKM values of floral organ identity and floral symmetry genes across three replicates for each organ in *R. coccinea*. Supplemental Data Set S4. Statistical summary of organ-specific and preferential gene expression in *R. coccinea*. Supplemental Data Set S5. Mean FPKM values of 37 genes specifically or preferentially expressed in the lip across three biological replicates at small and middle stages of *R. coccinea*. Supplemental Data Set S6. Mean metabolite abundance of 1158 compounds across three replicates for two organs in *R. coccinea*. Supplemental Data Set S7. Identification of differentially accumulated metabolites (DAMs) between small and middle stages in ds and ls samples. Supplemental Data Set S8. Mean FPKM values of anthocyanin biosynthetic pathway genes across three replicates for each organ in *R. coccinea*. Supplemental Data Set S9. Comparative expression profiling between ls samples between small and middle stages of *R. coccinea* by DESeq2. Supplemental Data Set S10. Comparative expression profiling between ds and ls samples at the middle stages of *R. coccinea* by DESeq2. Supplemental Data Set S11. Calculation of Pearson Correlation Coefficients (PCC) between the target gene and 39,390 other genes, emphasizing length classes and layout weights.
