# Supplementary material for: Integrative Multi-Omics Analysis Provides Insights into Floral Organ Development and Flower Color Formation in Renanthera coccinea, a Rare Tropical Orchid
Source: Biology (Basel). 2026 Sep 2;15(17):1497. doi: 10.3390/biology15171497 (PMC13565354; doi:10.3390/biology15171497)
Supplement: Supplementary file 1 [file biology-15-01497-s001.zip › Supplemental Figure S1-S10.pdf]

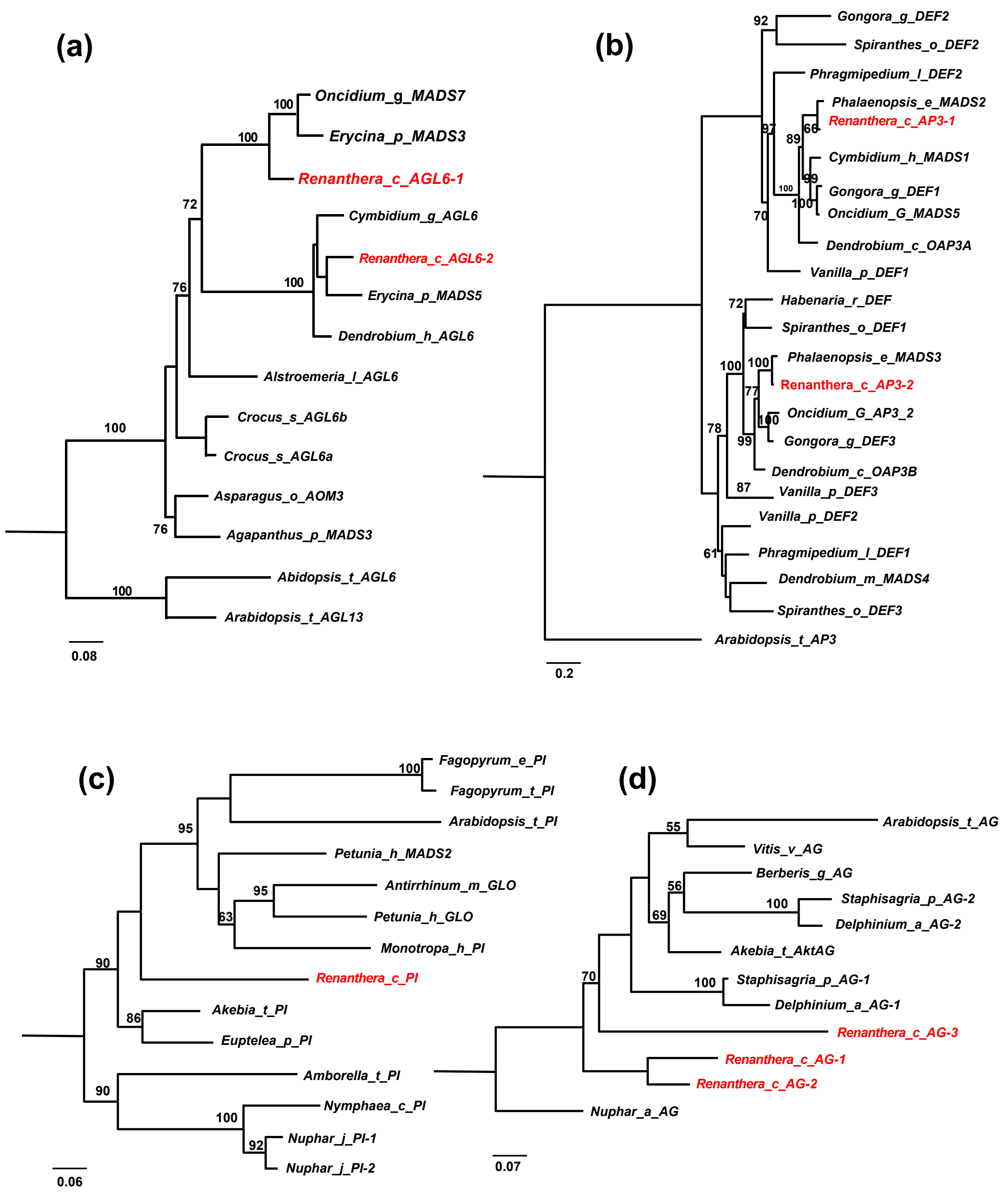

Supplemental Figure S1. Phylogenetic trees of floral organ identity genes.(a), *AGL6*-like genes. (b), *AP3*-like genes. (c), *PI*-like genes. (d), *AG*-like genes. Red, *Renanthera coccinea*; black, other lineages. Numbers associated with nodes are bootstrap values (≥ 50%). Branch lengths represent the number of nucleotide substitutions per site.

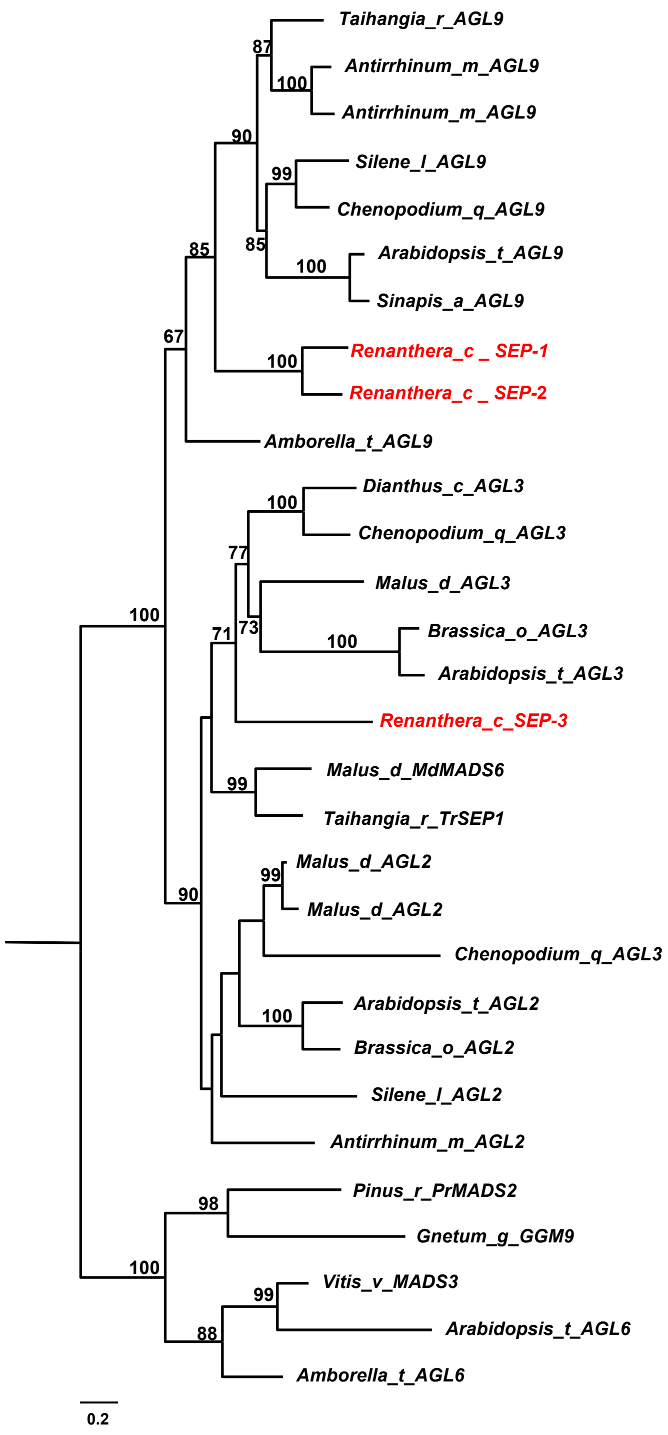

Supplemental Figure S2. Phylogenetic trees of *SEP*-like genes. Red, *Renanthera coccinea*; black, other lineages. Numbers associated with nodes are bootstrap values (≥ 50%). Branch lengths represent the number of nucleotide substitutions per site.

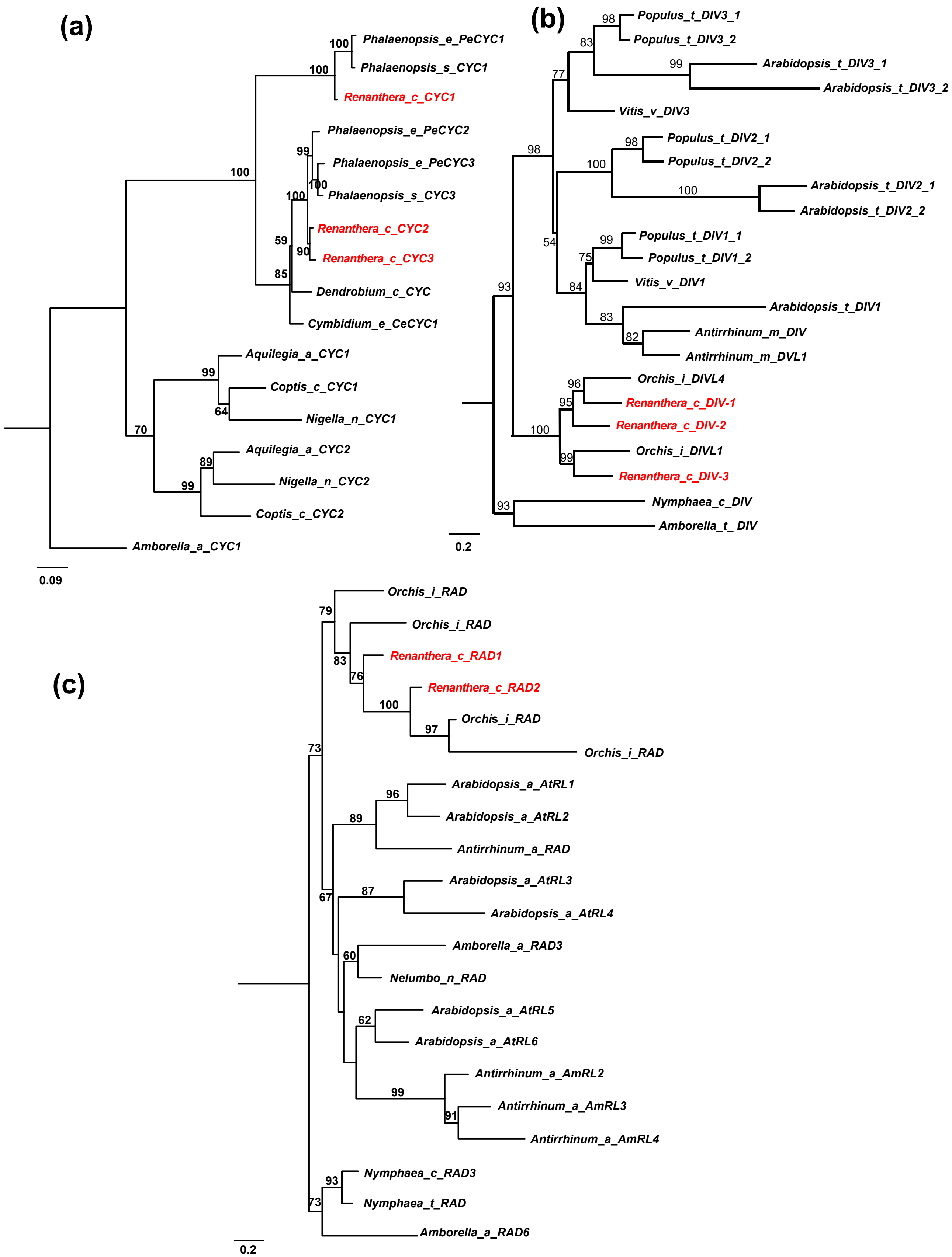

Supplemental Figure S3. Phylogenetic trees of floral symmetry genes genes. (a), *CYC*-like genes. (b), *DIV*-like genes. (c), *RAD*-like genes. Red, *Renanthera coccinea*; black, other lineages. Numbers associated with nodes are bootstrap values (≥ 50%). Branch lengths represent the number of nucleotide substitutions per site.

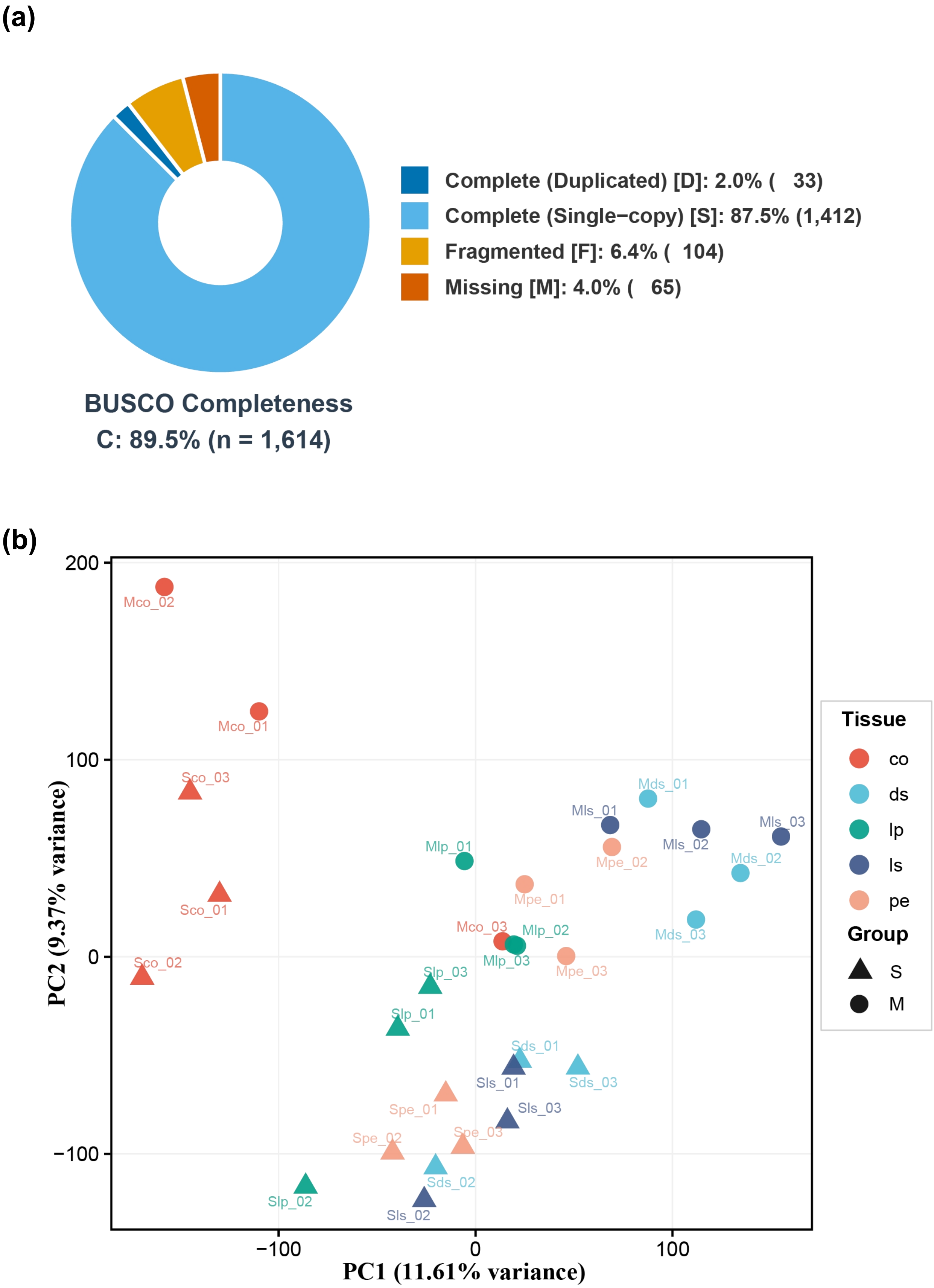

Supplemental Figure S4. Quality assessment and principal component analysis of transcriptomic data. (a) BUSCO assessment of transcriptome completeness (89.5%) ; (b) principal component analysis (PCA) of transcriptomic profiles across different tissues and treatment groups (n = 3 independent biological replicates).

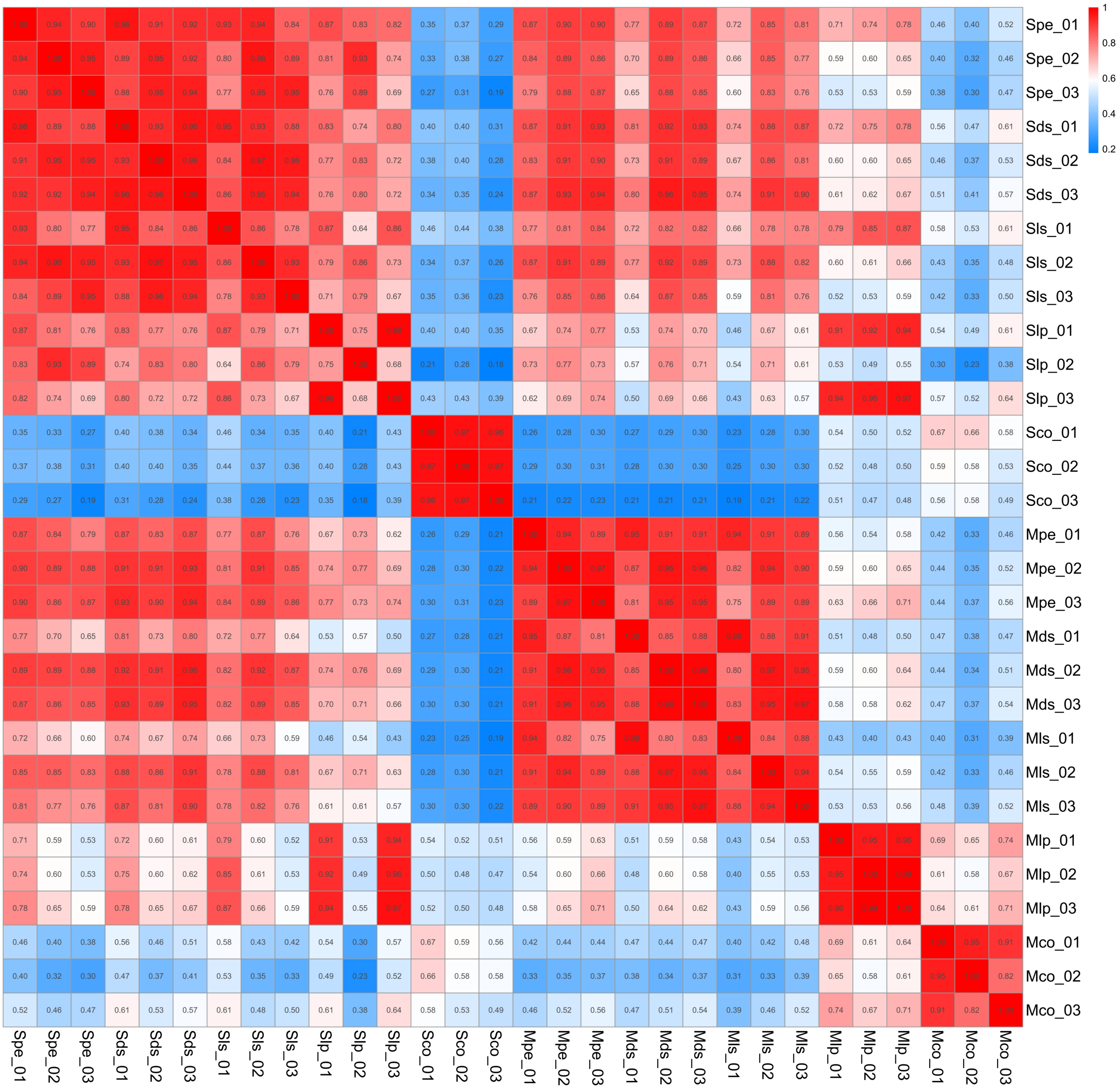

Supplemental Figure S5. sample correlation analysis of transcriptomic data in *R. coccinea*. (n = 3 independent biological replicates).

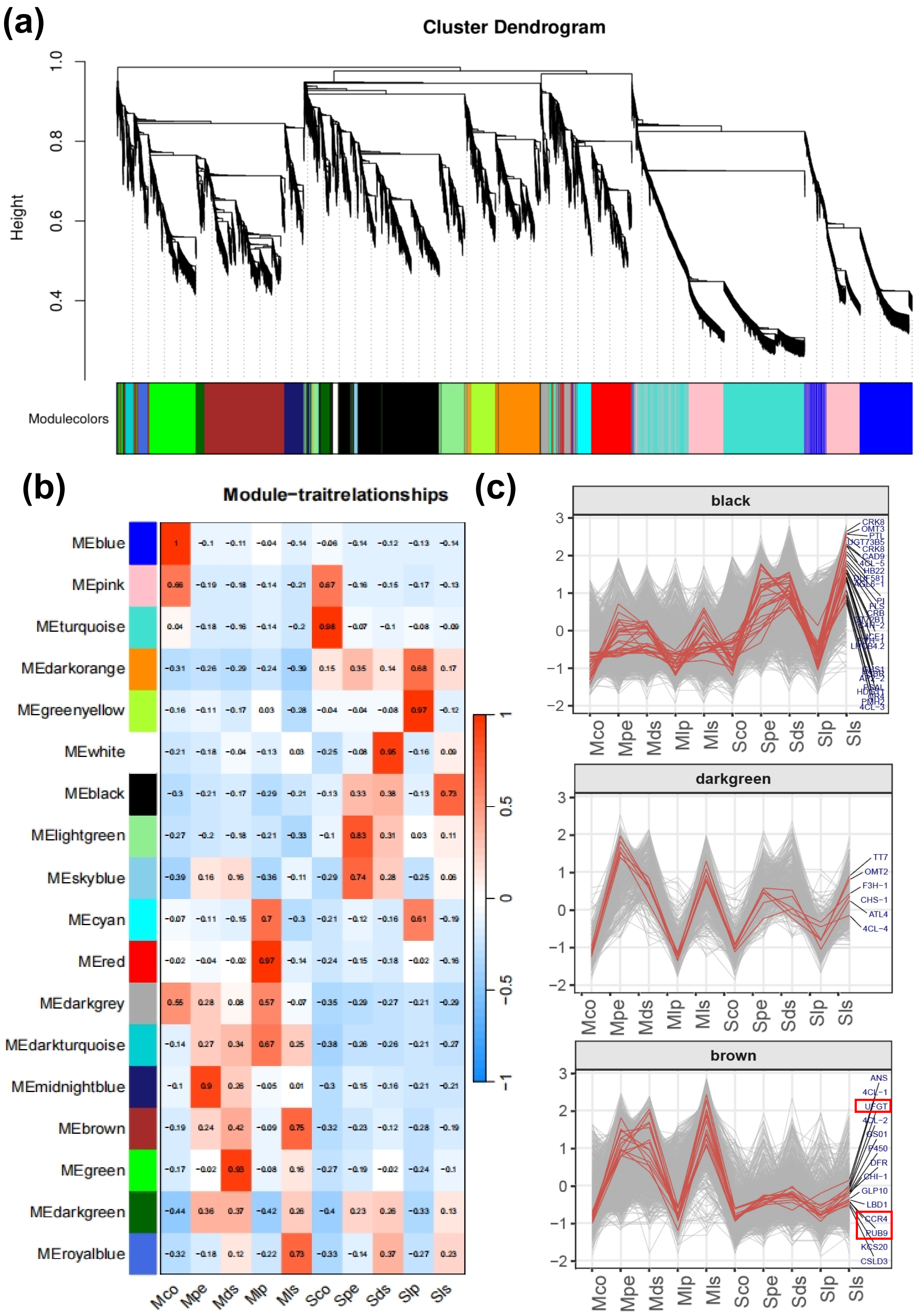

Supplemental Figure S6. Weighted gene co-expression network analysis (WGCNA) and expression profiles of key candidate modules.(a), Hierarchical clustering dendrogram showing identified co-expression modules based on topological overlap, with color bars below indicating module assignments. (b), Heatmap of module–sample relationships displaying Pearson correlation coefficients between module eigengenes (MEs, y-axis) and tissue samples across different developmental stages (x-axis, Mco–Sls). Color scale (red to blue) and cell values indicate correlation coefficients (red: positive correlation, blue: negative correlation). (c), Expression profiles of genes within key selected modules (black, darkgreen, and brown). Gray lines represent expression trajectories of all genes within the module, while red lines and blue labels highlight core/hub genes (e.g., *CHS-1*, *ANS*, *DFR*, and *UFGT*) (n = 3 independent biological replicates).

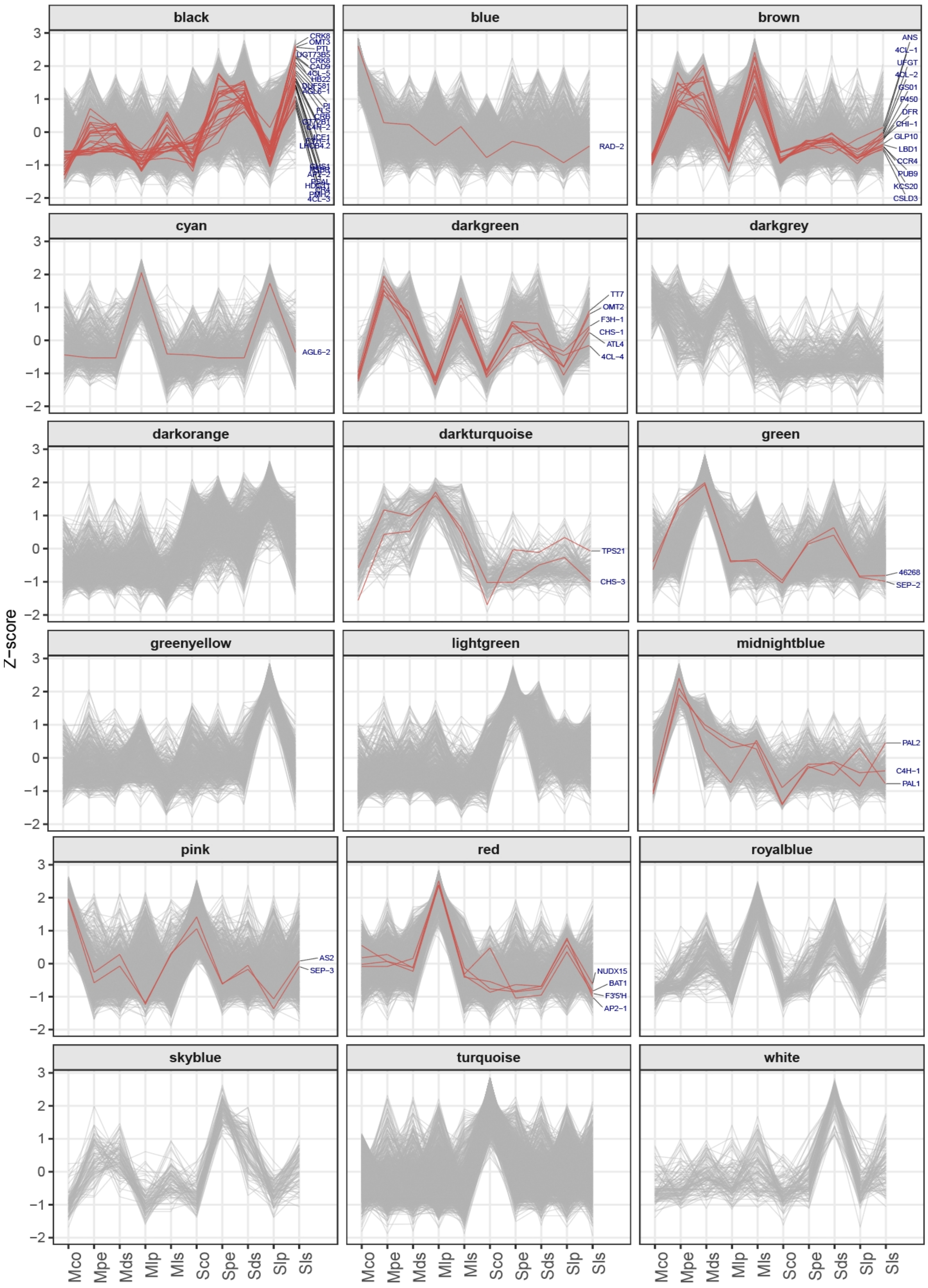

Supplemental Figure S7. Expression profiles of genes in WGCNA co-expression modules across different tissue samples. Expression trends of genes across 18 co-expression modules are depicted, with the x-axis representing 10 tissues (Mco, Mpe, Mds, Mlp, Mls, Sco, Spe, Sds, Slp, and Sls) and the y-axis indicating Z-score normalized relative expression levels. Gray lines represent the expression patterns of all genes within each module, whereas red lines with blue labels highlight key or hub genes of interest in specific modules (e.g., *ANS*, *DFR*, and *CHS-1*).

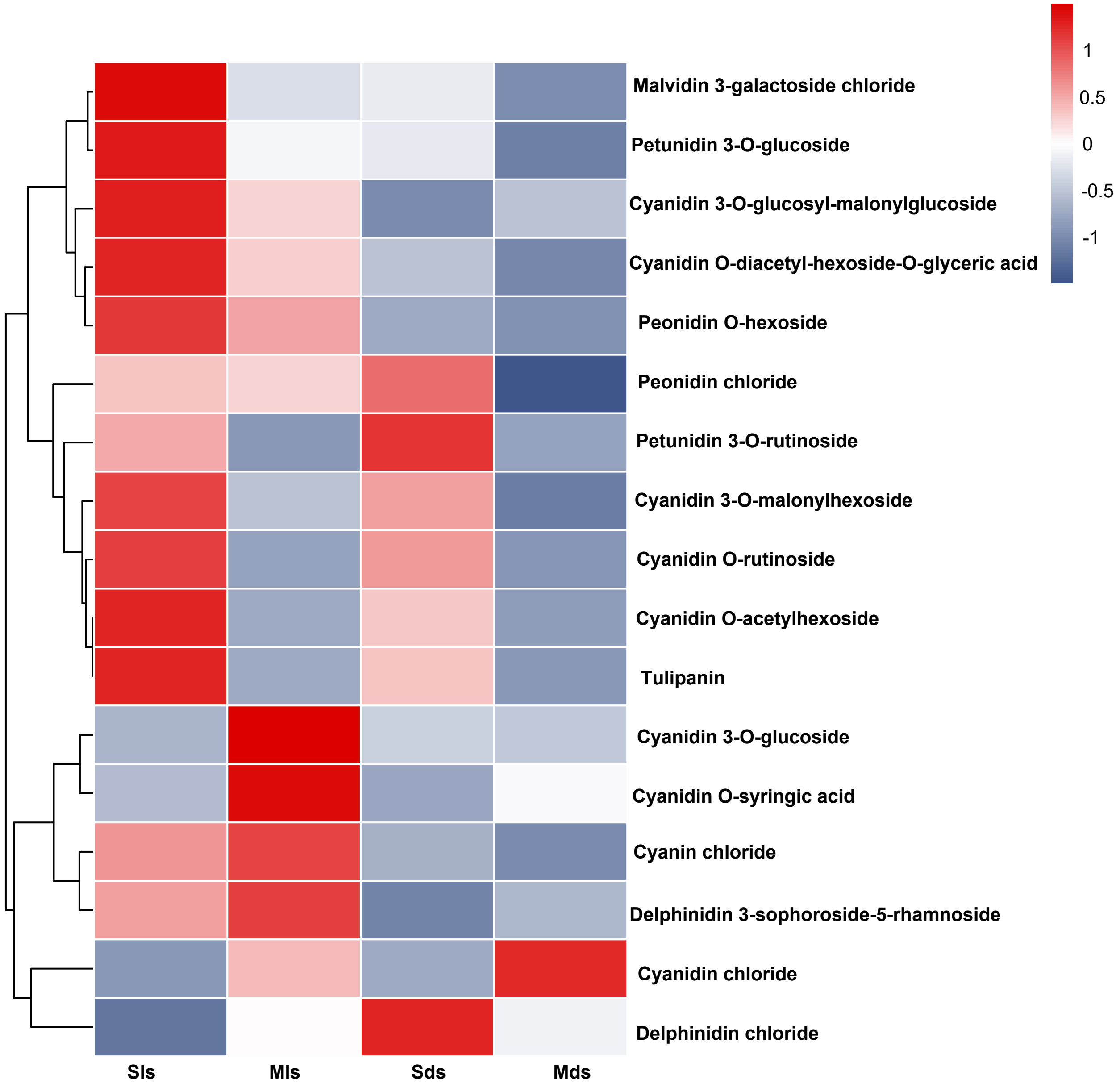

Supplemental Figure S8. Heatmap of 17 anthocyanin compounds in *R. coccinea* (n = 3 independent biological replicates).

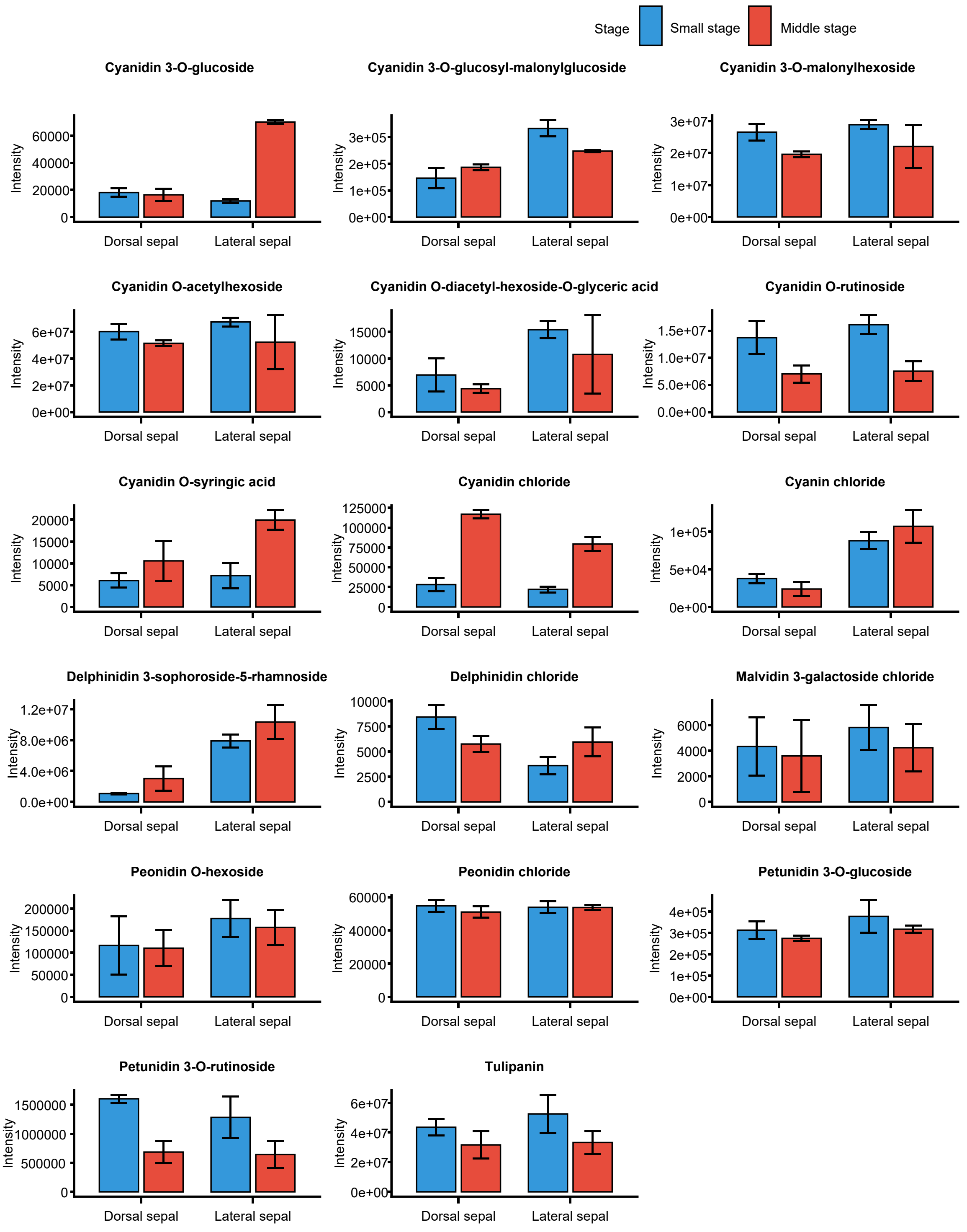

Supplemental Figure S9. Bar chart of 17 anthocyanin compounds in *R. coccinea* (n = 3 independent biological replicates).

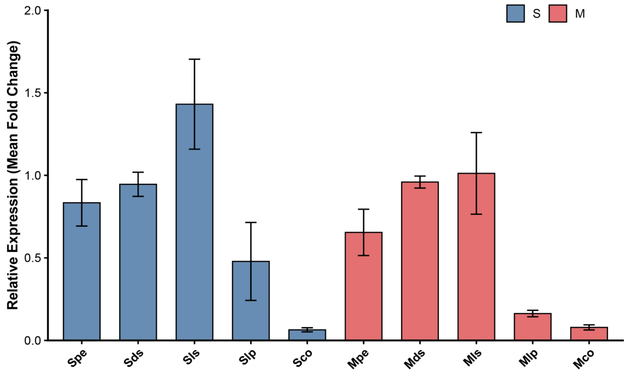

AGL6-1

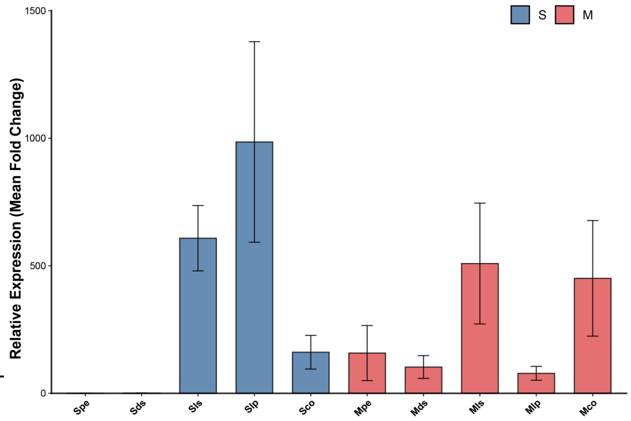

AGL6-2

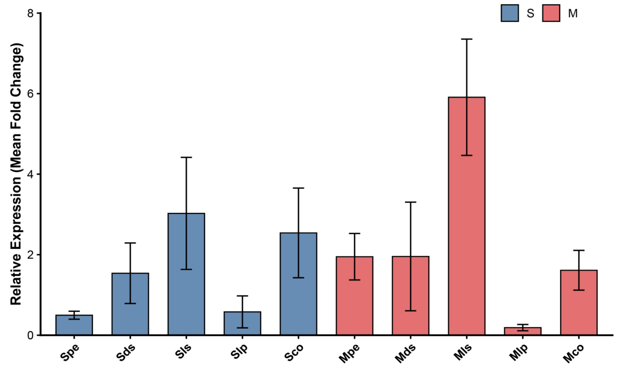

AP3-1

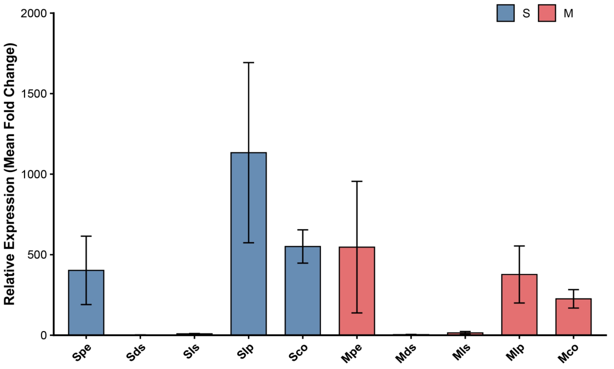

AP3-2

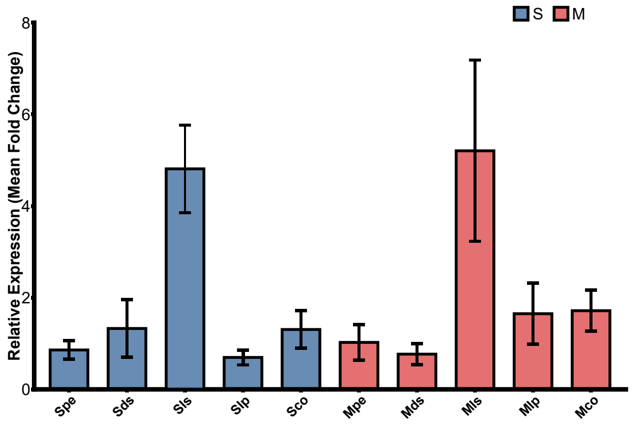

UFGT

Supplemental Figure S10. Relative expression levels of key candidate genes determined by qRT-PCR in *R. coccinea*.The relative expression of five candidate genes (*AGL6-1*, *AGL6-2*, *AP3-1*, *AP3-2*, and *UFGT*) was quantified using the $2^{-\Delta\Delta Cq}$ method in various tissue samples at the small stage (blue bars) and middle stage (red bars). Y-axes represent fold changes in relative expression levels, and data are presented as the mean ± SD (n = 3 independent biological replicates).
